# Decoding channel pore dynamics mechanism in *Anopheles gambiae* Orco homotetramer

**DOI:** 10.1093/bioadv/vbag295

**Published:** 2026-10-06

**Authors:** Vaanathi Chidambara Thanu, Amara Jabeen, Georgia Kythreoti, Kostas Iatrou, Spyros E Zographos, Philip W Taylor, Shoba Ranganathan

**Affiliations:** Applied BioSciences, Macquarie University, Sydney, NSW 2109, Australia; Applied BioSciences, Macquarie University, Sydney, NSW 2109, Australia; Department of Natural Sciences - Deree, The American College, Athens 15342, Greece; Institute of Biosciences & Applications, National Centre for Scientific Research “Demokritos”, Athens 15341, Greece; Institute of Biosciences & Applications, National Centre for Scientific Research “Demokritos”, Athens 15341, Greece; Institute of Chemical Biology, National Hellenic Research Foundation, Athens 11635, Greece; Applied BioSciences, Macquarie University, Sydney, NSW 2109, Australia; Applied BioSciences, Macquarie University, Sydney, NSW 2109, Australia

## Abstract

**Motivation:**

Insect odorant receptors (iORs) essential for olfaction function as 7-transmembrane-domain (7TM) ion channels. iORs form tetrameric complexes that include a highly conserved odorant receptor co-receptor (Orco). Functional Orco homotetramers have also known to form *ex vivo*. Blocking Orco *ex vivo* and *in vivo* disrupts olfactory signalling, making it a promising target for functional intervention. Understanding its gating behaviour is critical for developing strategies to disrupt odour-guided behaviours in disease-vector insects. Thus, insights into the dynamic gating of the *Anopheles gambiae* Orco have implications on the rational design of vector-control strategies against malaria. We studied molecular dynamics (MD) simulations on homotetrameric *Agam*Orco models generated using template-based modelling (TBM) and AlphaFold3 (AF3), to investigate ligand-dependent pore dynamics. Agonist and antagonist bound systems were analysed to understand the ligand-dependent gating.

**Results:**

Our results show that *apo* and antagonistic IPC-bound *Agam*Orco homotetramer models remain in a stable, closed, and non-conductive state across both models. TBM, but not AF3, successfully captures agonistic VUAA1-induced activation, consistent with experimental observations, exhibiting asymmetric pore widening and conformational changes. Antagonist displacement simulations confirm IPC-mediated pore closure. Overall, the TBM *Agam*Orco model provides a reliable framework for structure-guided discovery of novel agents for control of insect pests and vector diseases.

## 1 Introduction

Insects rely heavily on olfaction (Fleischer *et al.* 2018) to navigate their environment, guiding essential behaviours such as host seeking, mating, and oviposition. These processes are mediated by odorant receptors (iORs) (Francia and Lodovichi 2021), a large and diverse family of chemosensory proteins expressed in olfactory sensory neurons. In contrast to vertebrate ORs, which are G protein-coupled receptors, iORs function as ligand-gated ion channels (Li *et al.* 2026) and exhibit an inverted membrane topology (N-terminus in, C-terminus out), belonging to the seven-transmembrane helix (7TM) protein family. Insects enhance their chemical sensitivity through heteromeric OR complexes that include a highly conserved odorant receptor co-receptor (Orco) (Jacquin-Joly 2024). Orco is universally expressed across insect species and is essential for proper receptor localization, membrane targeting, and channel activity (Butterwick *et al.* 2018, Zufall and Domingos 2018, Bobkov *et al.* 2021). Genetic disruption of Orco abolishes odour-evoked responses, underscoring its central role in olfactory signalling (Sun *et al.* 2020, Chen *et al.* 2025). This remarkable evolutionary conservation of Orco highlights its fundamental importance in insect chemosensation. Beyond its role as a co-receptor, Orco alone may also assemble as a homotetrameric (HT) complex capable of ion conduction independent of tuning ORs (Butterwick *et al.* 2018, Zufall and Domingos 2018). Synthetic agonists like VUAA1 (Vanderbilt University Allosteric Agonist One) [N-(4-Ethylphenyl)-2-((4-ethyl-5–(3-pyridinyl)-4H-1,2,4-triazol-3-yl)thio)acetamide] or its isomer OrcoRAM2 can activate Orco homomeric channels *ex vivo*, demonstrating the ability to function independently of the tuning OR subunit (Tsitoura *et al.* 2015, Tsitoura and Iatrou 2016). The cryo-electron microscopy (cryo-EM) structure of the *Apocrypta bakeri* Orco (*Ab*Orco) shows a tetrameric ligand-gated ion channel with pinwheel-like architecture (Butterwick *et al.* 2018). Each subunit contains seven transmembrane helices (*S1-S7*), with *S1-S6* forming an extracellular ligand-binding pocket and terminal part of the seventh helix (*S7b*) from each subunit lining the central ion-conduction pore. The pore is constricted near the extracellular side by conserved hydrophobic residues that stabilize the closed state of the channel. VUAA1 activates the *Ab*Orco ion channel with dynamic pore opening, possibly associated with conformational changes. Such transient events are difficult to capture experimentally and cannot be fully characterized using static structural snapshots alone. To overcome this, researchers often combine these static structures with complementary methods to visualize the dynamic transitions. Molecular dynamics (MD) simulations, therefore, provide a powerful framework to investigate the pore dynamics (Tiwari and Sowdhamini 2023, Xue *et al.* 2025), while enhanced sampling approaches like Gaussian accelerated Molecular dynamics GaMD (Miao *et al.* 2015, Wang *et al.* 2021) allow exploration of conformational transitions that occur on timescales beyond those typically accessible to conventional MD (cMD) simulations.

*Anopheles gambiae* is the principal vector of malaria, one of the most significant global health challenges worldwide (Li *et al.* 2021). As *A. gambiae* has developed resistance to many chemicals (Azizi *et al.* 2024), vector control has become more challenging. Understanding Orco function is critical for vector-control strategies as disruption of Orco function could reduce host-seeking behaviour, thereby limiting disease transmission. *Ex vivo* studies of the functional *A. gambiae* Orco (*Agam*Orco) HT (Tsitoura *et al.* 2015, Tsitoura and Iatrou 2016) demonstrated that inhibition of mosquito odorant receptor function occurs through efficient Orco blocking, suggesting that repellents might disrupt olfactory signalling by interfering with Orco-mediated channel gating or ion conduction. Furthermore, a recent experimental study (Kythreoti *et al.* 2024) investigated Orco orthosteric antagonists and their role in disrupting mosquito behaviour, even in the presence of agonists. A study of the fruitfly, *Drosophila melanogaster* Orco HT (*Dmel*Orco) (Pacalon *et al.* 2023) identified the agonist binding site, explaining the high selectivity of Orco for its ligands. Together, these findings highlight Orco as an ideal candidate to computationally study the channel pore and dynamic gating, with different ligands. Understanding the intrinsic gating behaviour of Orco, in its HT form, is critical for deciphering fundamental principles of insect olfaction and for developing strategies to disrupt odour-guided behaviours in disease-vector insects. Insights into the dynamic gating and pore-opening mechanisms of *Agam*Orco therefore have direct implications for the rational design of novel vector-control strategies against malaria.

In this work, we compare *Agam*Orco HT bound to an agonist, VUAA1 and an antagonist, isopropyl cinnamate (IPC), to determine how agonist and antagonist binding differentially influence channel pore conformations. In the absence of a cryo-EM HT structure for *Anopheles gambiae* or any other mosquito species, we developed a template-based model (TBM) of *Agam*Orco HT using SWISS-MODEL (Waterhouse *et al.* 2018), with *Ab*Orco serving as starting model. The TBM HT model was examined for its stability and pore dynamics in the *apo* and *holo* states with bound VUAA1 or IPC, using MD simulations. AlphaFold3 (AF3) (Abramson *et al.* 2024) can predict structures from input polymer sequences for multimeric proteins. We have therefore compared our TBM models with AF3 models, to check which models can accurately display the experimentally verified functional characteristics (Tsitoura *et al.* 2015, Tsitoura and Iatrou 2016, Kythreoti *et al.* 2024).

We performed simulations with varying agonist-antagonist ratios, including equal and biased compositions, to determine the minimum antagonist: agonist stoichiometry. Furthermore, to study the effects of ligand stoichiometry and to assess whether antagonists can reverse or stabilize closure of an agonist-induced open pore, simulations were initiated from agonist-opened pore conformation by replacing agonists with antagonists, alongside ligand-free open-pore controls. Pore dynamics were characterized through pore radial profile analysis, inter-chain gate distance analysis, dynamics of key pore-lining residues within the *S7b* helix, and ligand binding interactions. Changes in pore geometry provided structural evidence for ligand-specific gating behaviour that could not be inferred from ligand binding interactions alone. Overall, this comprehensive approach, combining structural stability metrics, pore dynamics, and ligand interactions, offers detailed insights into the structural and functional changes in *Agam*Orco induced by ligand binding, and provides mechanistic insights into how ligand identity and stoichiometry influence Orco pore gating and stability, advancing our understanding of the molecular mechanisms governing odorant receptor gating. These insights enable structure-guided discovery of Orco modulators with likely applications also for insect repellents acting on heteromeric iORs, and represent the first ligand-dependent, MD-based dynamic characterization of pore opening and stability in *Agam*Orco HT, linking ligand binding to functional ion-channel states.

## 2 Methods

*Agam*Orco sequence was retrieved in FASTA format from the UniProt database (Ahmad *et al.* 2025). The best structural template for TBM was identified using iBio-GATS (Chidambara Thanu *et al.* 2024), a semi-automated workflow for iOR modelling, by searching available experimental structures. The selected template structure was downloaded from the Protein Data Bank (PDB) (Berman *et al.* 2000). Orco agonist VUAA1 and antagonist IPC (Supplementary Fig. S1) structures were retrieved in SDF format from Pubchem (Kim *et al.* 2025) and subsequently converted into PDB format using UCSF ChimeraX (v1.4) (Pettersen *et al.* 2021).

iBio-GATS currently generates monomeric iOR models (Chidambara Thanu *et al.* 2025). Based on the iBioGATS-selected template of *Ab*Orco, HT models of *Agam*Orco were generated using SWISS-MODEL via the ExPASy web server (Waterhouse *et al.* 2018), with the top model selected based on the SWISS-MODEL quality metrics. In addition, the AF3 HT *Agam*Orco model was generated using the AF3 web server (Abramson *et al.* 2024), and the top-ranked model was selected based on confidence metrics. In addition, a complete HT *Ab*Orco model was built using SWISS-MODEL, with the experimental structure (PDB ID: 6C70) as template, as a reference for channel pore dimensions and conformational changes, as the 6C70 structure, lacking two long loops, is unstable under MD simulation conditions.

Binding pockets for each model were identified based on previously reported site-directed mutagenesis and functional studies of *Dmel*Orco (Pacalon *et al.* 2023). The docking setup was as described earlier (Chidambara Thanu *et al.* 2025). Grid-based docking was performed using AutoDock Vina 1.2.0 (Eberhardt *et al.* 2021), with VUAA1 and IPC, for each HT chain, and the top-scoring pose for each complex was selected based on the strongest binding affinity (kcal/mol). Protein–ligand binding interactions were visualized and analysed using BIOVIA Discovery Studio Visualizer (Dassault Systemes 2021) and UCSF Chimera (v1.16) (Pettersen *et al.* 2004).

cMD simulations were performed to refine the *Agam*Orco HT in *apo*, agonist-bound, and antagonist-bound states within a membrane environment and to investigate the conformational stability and dynamics using Amber 22 (Case *et al.* 2022). Membrane systems were constructed with CHARMM-GUI Membrane Builder (Wu *et al.* 2014). Supplementary Note S1 describes membrane composition, the MD setup and process in detail.

GaMD simulations (Wang *et al.* 2021) were performed using an open-pore structure selected from the equilibrated cMD trajectory between 900 ns and 1 µs as the starting conformation. Supplementary Note S2 describes the GaMD setup and protocol followed. The resulting GaMD trajectories were used to analyse conformational changes associated with channel gating and ligand-dependent changes in pore architecture. Pore behaviour during production was assessed by monitoring gate distances and channel analysis. These analyses were used to check whether the agonist-bound system can maintain or sample open pore conformations in either symmetric or asymmetric state.

The Molecular Mechanics Poisson–Boltzmann Surface Area (MM/PBSA) (Miller *et al.* 2012) and Molecular Mechanics Generalised Born Surface Area (MM/GBSA) (Forouzesh and Mishra 2021) binding free energies (ΔG_bind_) in kcal mol^−1^ were estimated post-dynamics, using AMBER’s CPPTRAJ module (Roe and Cheatham 2013).

CPPTRAJ was also used for root mean square deviation (RMSD) analysis to evaluate overall structural stability, root mean square fluctuation (RMSF) calculations for residue-level flexibility, radius of gyration (Rg) for global compactness and hydrogen bond analysis to evaluate ligand-induced changes in intra- and inter-chain interactions. Helix conformational dynamics were assessed from helix RMSD analysis. Principal component analysis (PCA) (Moradi *et al.* 2024) of the gate residues was performed to characterize dominant collective motions and conformational changes sampled during the simulations. The data were plotted with Python version 3.10.6 (Van Rossum and Drake 2009). All visualization was carried out using Virtual Molecular Dynamics (VMD) (Humphrey *et al.* 1996) and Discovery Studio. To characterize the pore geometry, inter-chain distances between equivalent pore-gate residues were calculated throughout the simulations using VMD. HOLE (Smart *et al.* 1996) was used to generate pore-radius profiles along the channel axis, with output visualized in PyMOL 3.1.6.1 (www.pymol.org) to produce 3D representations and to analyse variations in the pore radius along the channel axis. The minimum pore radius, corresponding to the bottleneck region of the pore, was monitored over time to track pore opening.

## 3 Results

The sequence identity between the *Ab*Orco template sequence from 6C70 and the target, *Agam*Orco, was found to be 66%. The TBM HT model (Supplementary Fig. S2a–c) shows a helical RMSD of 0.181 Å from the template. The TBM alignment generated by SWISS-MODEL is provided in Supplementary Fig. S3, with the helices numbered (S1–S7). The AF3 *Agam*Orco HT model (Supplementary Fig. S2d–f) has a helical RMSD of 0.731 Å, compared to the *Ab*Orco model. The initial inter-chain distances of the gate residues between chain pairs AC and BD (Fig. 1) in the TBM and AF3 *Agam*Orco models were comparable to those of the *Ab*Orco model (Supplementary Table S1), thereby underscoring the structural integrity and reliability of the models. The initial *Agam*Orco and the *Ab*Orco models in the space-filling view, along with the inter-chain distance measurement for the gate residue Leu477 in *Agam*Orco and the corresponding Leu473 residue in the template, are given in Supplementary Fig. S4. Docking of VUAA1 with the TBM model (Supplementary Fig. S5), under saturating conditions captured all five key binding pocket residues: Phe81 (*S2*), Phe82(*S2*), Ser144(*S3*), Met208(*S4*), and Tyr392(*S6*) identified for *Dmel*Orco (Pacalon *et al.* 2023). In contrast, the *holo* AF3 model with VUAA1 (Supplementary Fig. S6) captured only three key residues, reflecting a slightly different interaction pattern. IPC occupied the same orthosteric pocket in both models (Supplementary Figs S7 and S8), with TBM capturing all five key residues while AF3 identified only four.

**Figure 1:**
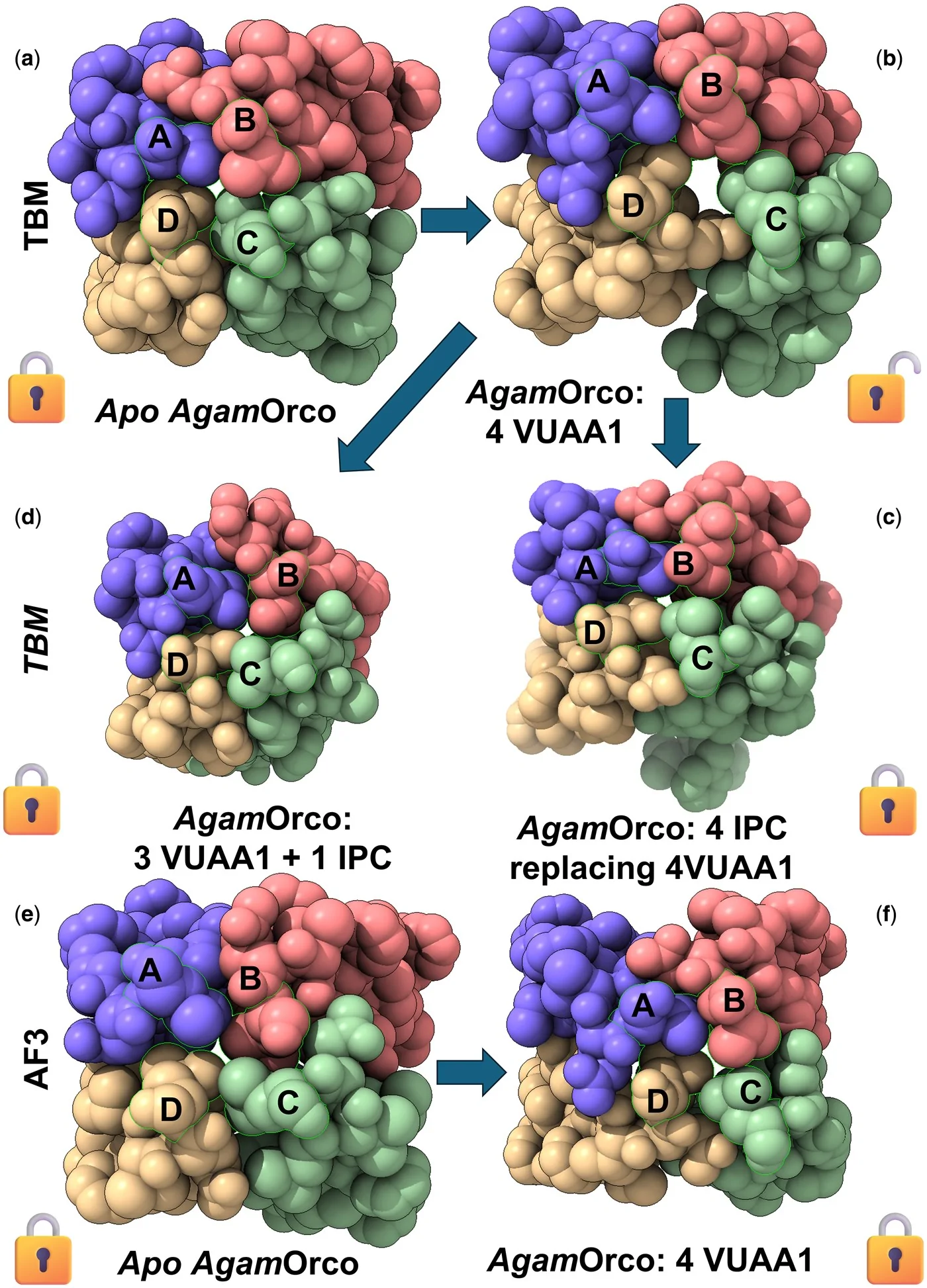
Channel pore dynamics of *Agam*Orco HT following 500 ns cMD simulations. (a) *apo* TBM *Agam*Orco, (b) 4 VUAA1-bound TBM *Agam*Orco, (c) 4 IPC: replacing 4VUAA1 in open pore conformation of TBM *Agam*Orco, (d) 3 VUAA1:1 IPC -bound TBM *Agam*Orco, (e) *apo* AF3 *Agam*Orco, and (f) VUAA1-bound AF3 *Agam*Orco. The channel pore status is indicated with a lock symbol. Tetramer chains are labelled A-D.

### 3.1 TBM pore channel opens only with agonist binding

Following 500 ns of cMD simulations (Fig. 1, Supplementary Fig. S9), all *apo* models retained a closed channel pore. With four VUAA1 agonist molecules bound (saturating conditions), the TBM *Agam*Orco model pore opens, as reported experimentally (Fig. 1b) (Tsitoura *et al.* 2015, Tsitoura and Iatrou 2016), similar to *Ab*Orco (Supplementary Fig. S9b) while the AF3 *Agam*Orco model pore remains closed (Fig. 1e and f, Supplementary Fig. S9c). With four bound IPC antagonist molecules, the channel pore remained closed in both TBM and AF3 *Agam*Orco models, as expected (Supplementary Fig. S9).

Consistent pore expansion is observed across the gate residues (Val473-Leu477) along the AC axis, which exhibits initial expansion followed by stabilization, indicating maintenance of an expanded pore along this axis. The BD axis remained relatively stable or showed minor fluctuations between 300 and 500 ns, indicating that the pore stabilized with a slight decrease in the distance along the BD axis while maintaining the expansion along AC, suggesting that the pore stabilized in an asymmetric open state rather than a symmetric open conformation even after GaMD simulations (Supplementary Tables S2 and S3). Distance changes are shown in Fig. 2, for *Agam*Orco TBM and AF3 models, compared with the *Ab*Orco model. PCA of the gate residues of TBM *Agam*Orco models over the 500 ns cMD simulations (Supplementary Fig. S10) revealed distinct conformational sampling across the *apo*, agonist-bound, and antagonist-bound states. PC1 and PC2 explained 36.2% and 29.7% of the total variance, respectively. The *apo* state formed a relatively compact conformational cluster, whereas the antagonist-bound state occupied a distinct but slightly broader region of conformational space. In contrast, the agonist-bound state showed a markedly broader and multimodal distribution, suggesting that the pore-lining residues sampled multiple conformational substates during the simulation. Overall, these differences indicate ligand-dependent changes in pore conformational dynamics.

**Figure 2:**
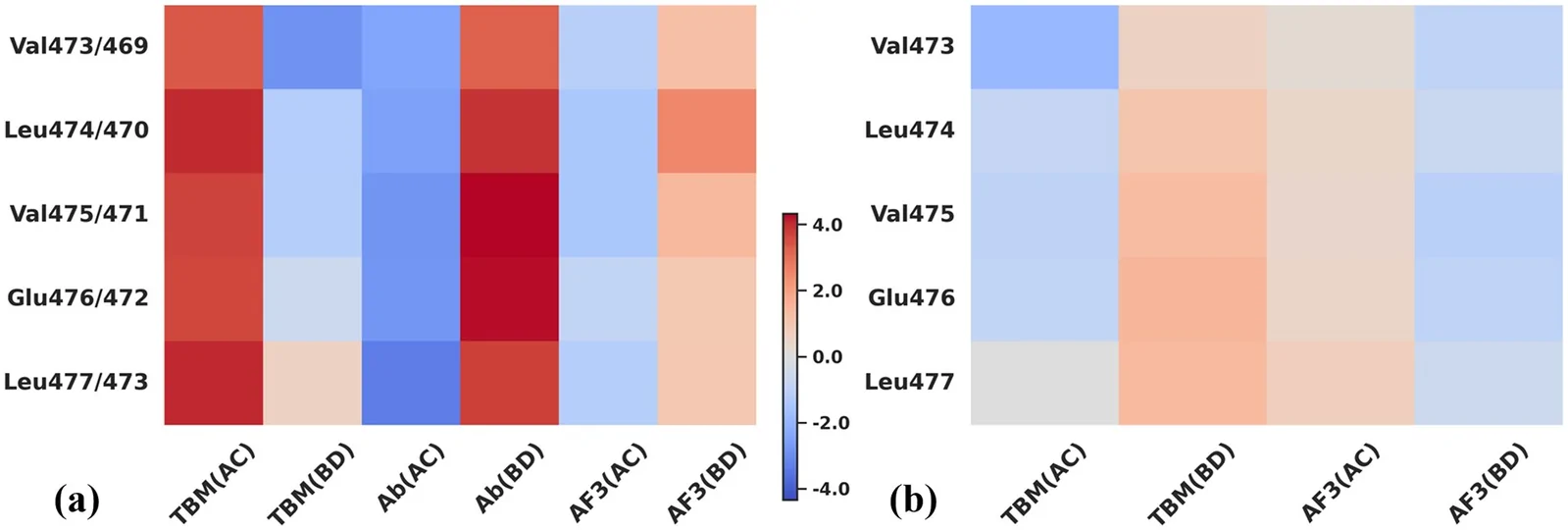
Distance changes in the gate residues along the AC and BD axes after 500 ns cMD simulations upon ligand binding. (a) *Ab*Orco (Ab), TBM *Agam*Orco (TBM), and AF3 *Agam*Orco (AF3) models upon VUAA1(agonist) binding, and (b) TBM *Agam*Orco, and AF3 *Agam*Orco models upon IPC (antagonist) binding.

All HT models were stable throughout the 500 ns cMD simulations (Supplementary Fig. S11). The experimental template structure of HT AbOrco (PDB: 6C70) started to unravel during cMD simulations, necessitating the building of the complete HT AbOrco models for this study. RMSD, RMSF and Rg analyses are available in Supplementary Note S3 and Supplementary Figs S12–S15. Extension of the cMD simulation to 1 µs confirmed that the TBM *Agam*Orco complex with bound agonist VUAA1 remained open after 500 ns, in an asymmetric conformation. Subsequent GaMD simulations sampled larger pore radii and clearly showed asymmetrical expansion with more pronounced and sustained widening of the central constriction. In particular, the pore sampled a broader range of radii, with increased inter-chain distances along both the AC and BD axes, especially at Gln476 (Supplementary Fig. S16) and Leu477 (Supplementary Table S3), reflecting increased flexibility and frequent transient opening events. In contrast, the AF3 *Agam*Orco-VUAA1 complex showed only minor fluctuations in pore distances and remained functionally closed throughout the 500 ns simulation. Also, both TBM and AF3 models bound to the antagonist IPC showed little to no change in inter-chain pore distances, indicating the absence of significant pore opening (Supplementary Table S2).

Channel pore analysis revealed that both HT TBM and AF3 *apo Agam*Orco maintained a narrow central pore, with minimum radii of around 0.52–0.57 Å for TBM and 0.97–1 Å for AF3, consistent with the closed state of the *apo Ab*Orco model (Fig. 3). The constriction remained below 1 Å throughout the 500 ns simulation, indicating a functionally closed hydrophobic gate, impermeable to water or ions.

**Figure 3:**
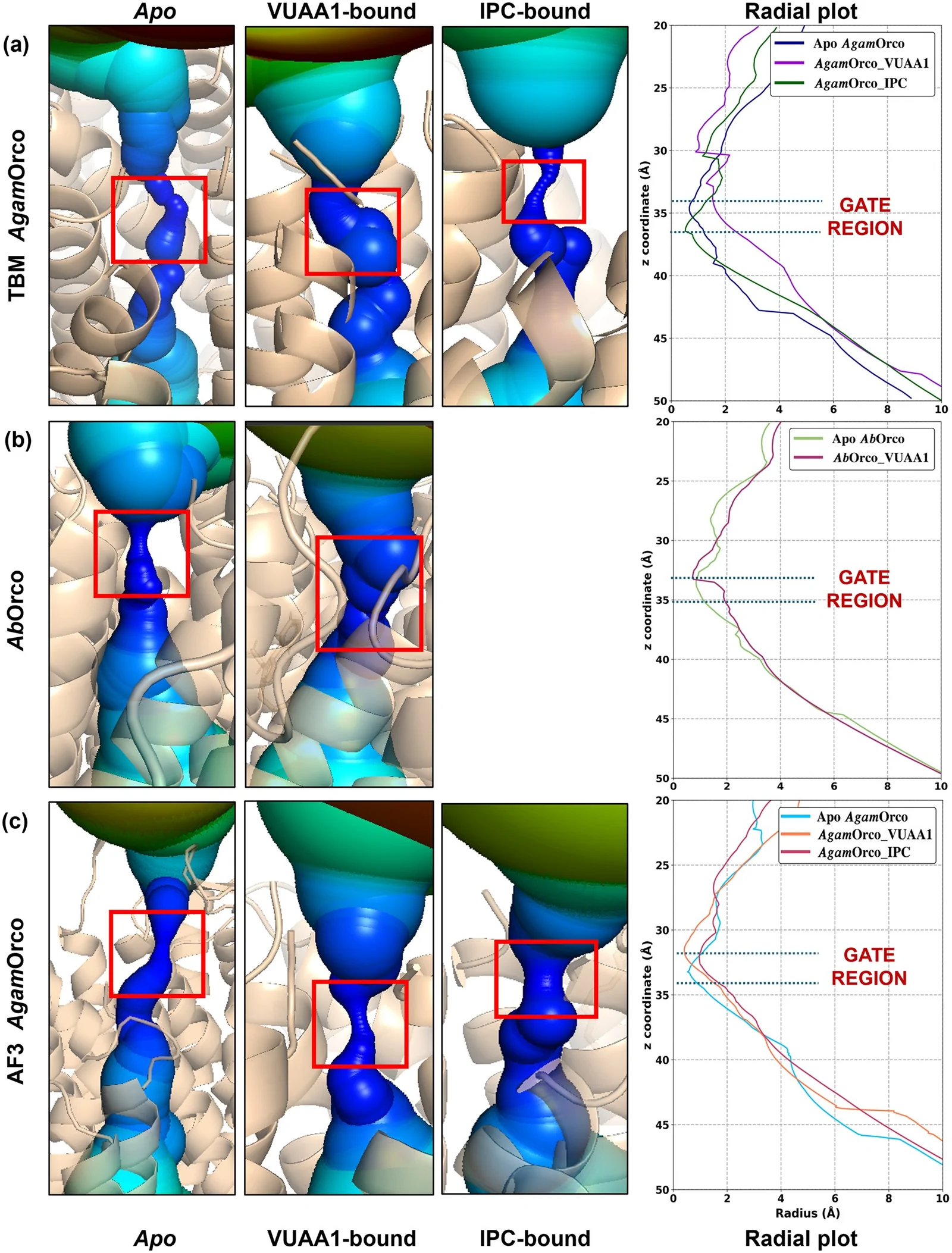
Channel pore and radial profile after 500 ns cMD simulations. (a) *apo*, VUAA1-bound, and IPC-bound models of TBM *Agam*Orco, (b) *apo* and VUAA1-bound models of *Ab*Orco, and (c) *apo*, VUAA1-bound, and IPC-bound models of AF3 *Agam*Orco. The *holo* models have four ligand molecules bound. The gate is highlightedwith a rectangle.

Pore visualization confirmed the persistent central constriction in both apo models (Fig. 3). Upon VUAA1 binding, the TBM model displayed pronounced widening of the central pore, with minimum radii increasing to around 1.5–2 Å at 500 ns, consistent with asymmetric pore opening observed in inter-chain distance analyses and comparable to the VUAA1-bound *Ab*Orco (Fig. 3). In contrast, the AF3-VUAA1 complex retained a narrow pore (∼1 Å), indicating no substantial opening, with both radial profiles and visualization showing a compact, closed pore. For IPC-bound complexes, the pore radius of TBM remained approximately 1–1.2 Å, and AF3 approximately 1 Å at 500 ns, showing no significant widening compared with *apo* states. The overall channel pore was stable, indicating that IPC binding did not induce pore opening in either TBM or AF3 models (Fig. 3, Supplementary Fig. S9). Post-MD analysis of the ligand binding pockets is presented in Supplementary Figs S17–S20, detailing residues stabilizing both agonist and antagonist in the orthosteric ligand-binding pocket. Mean helical RMSD differences (Supplementary Table S4) between the initial (1 ns) and final (500 ns) conformations of TBM *Agam*Orco complexes revealed ligand-dependent conformational changes. The *apo* system showed stable helical conformations with limited rearrangement, whereas VUAA1 binding induced larger deviations in helix *S7b* (2.8 Å, Chain C) and helix *S4* (2.1 Å, Chain C), and a moderate deviation in helix *S6* (1.7 Å, Chain A). In contrast, IPC binding resulted in relatively smaller helix deviations, with only moderate changes in helix *S6* (1.6 Å, Chain D), supporting a stable closed channel pore conformation. Helix tilt (Supplementary Table S5) analysis in *S7b* helices showed no major differences between *apo*, agonist-bound, and antagonist-bound complexes, suggesting that channel activation is not primarily driven by large changes in helix inclination. In contrast, helix reorientation analysis (Supplementary Table S5) revealed a pronounced ligand-dependent rearrangement, with agonist-bound *S7b* showing substantial reorientation in Chain C (13.9°) compared with *apo* (0.7°) and antagonist-bound (1.0°) states, suggesting asymmetric reorientation of pore-associated helices during activation.

Also, hydrogen bond analysis (Supplementary Table S6) comparing the early (1–10 ns) and late (490–500 ns) phases of the agonist-bound simulations revealed changes of *S7b* inter- and intra-chain interactions during activation. Analysis of key hydrogen bond occupancy changes > 1% revealed the loss of Gln476(A)-Lys478(D) interaction together with the formation of a new Leu477(A)-Lys478(B) interaction. The intra-chain analysis revealed loss of the Leu474(B)-Lys478(B) interaction, compensated by a new Gln476(C)-Lys478(C) interaction.

Binding energy analysis showed that VUAA1 had favourable binding in both TBM and AF3 *Agam*Orco models, with negative GB and PB binding energies across all four chains after 500 ns, comparable to the *Ab*Orco template binding energies (Supplementary Table S7). However, the TBM model showed stronger overall binding energies than the AF3 model. In contrast, IPC binding was energetically more favourable in the TBM model than in AF3, though the binding energies of IPC were weaker than those observed for VUAA1 for both models (Supplementary Table S7).

### 3.2 Pore channel opens with agonist binding in TBM model and closes with antagonist addition

To analyse the displacement of the antagonist IPC on the agonist VUAA1, two simulations experiments were conducted, starting from a specific open-pore conformation from cMD simulation with bound agonist, and from GaMD simulation of VUAA1-bound *Agam*Orco HT, to simulate an open pore *apo* conformation.

The addition of IPC was able to close the open pore conformation (Fig. 1) and details are available in Supplementary Notes S4 and S5, Supplementary Figs S21–S23, and Supplementary Tables S8 and S9. In the cMD-derived system, the pore narrowed rapidly within the first 100–200 ns, approaching distances similar to the *apo* HT, indicating a transition to a more constricted state (Supplementary Table S8). In the GaMD-derived system, pore narrowing was more gradual, occurring primarily between 300–500 ns, without complete closure, suggesting that antagonist binding drives the pore towards a closed conformation over time, while the *apo* reference remained expanded throughout the 500 ns simulation (Supplementary Table S8).

Replacing the VUAA1 molecules in the agonist-bound complex systematically with IPC resulted in pore closure with a single antagonist (3 VUAA1: 1 IPC) (Fig. 1). The inter-chain distance analysis (Supplementary Table S10, Fig. 4) supports this finding. Details are available in Supplementary Note S6, Supplementary Fig. S24, and Supplementary Table S11, showing the *Agam*Orco pore behaviour varied with the ligand concentration, although none of these conditions resulted in pore opening. The antagonist-rich system (1 VUAA1: 3 IPC) exhibited greater fluctuations, the balanced system (2 VUAA1: 2 IPC) showed moderate fluctuations with slight narrowing, and the agonist-rich system (3 VUAA1: 1 IPC) showed initial fluctuations followed by pore narrowing rather than progressive opening. Overall, even under agonist-rich conditions, the pore remained dynamically fluctuating and did not stabilize into an open conformation.

**Figure 4:**
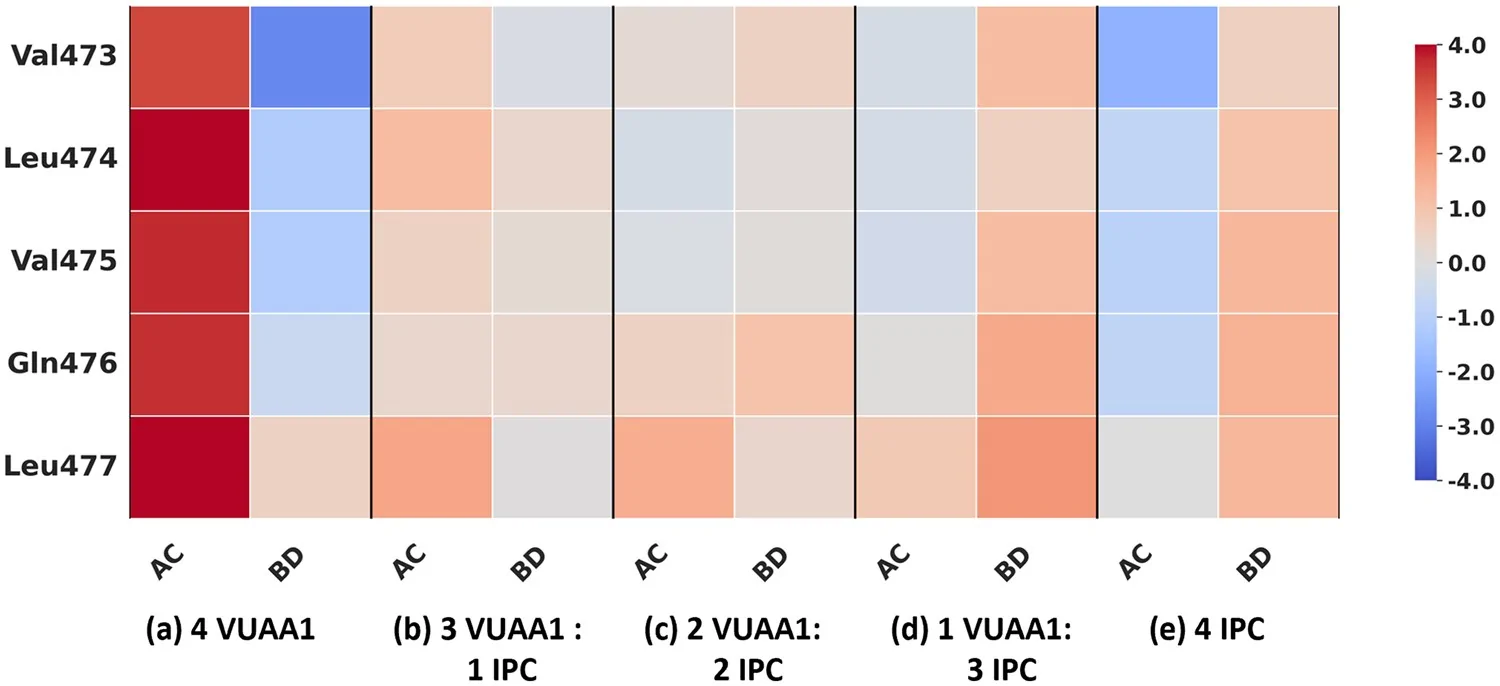
Distance changes in the gate residues of *Agam*Orco TBM models along the AC and BD axes after 500 ns cMD simulations, upon step-wise replacement of VUAA1 (agonist) by IPC (antagonist). TBM *Agam*Orco with (a) 4 VUAA1, (b) 3 VUAA1: 1 IPC, (c) 2 VUAA1: 2 IPC, (d) 1 VUAA1: 3 IPC, and (e) 4 IPC.

## 4 Discussion

We have shown that the TBM *Agam*Orco HT model captures structural features consistent with the consistent with the *ex vivo* functional evidence, supporting its application in structure-based antagonist design. The TBM model provides important clues for understanding the conserved pore architecture and gating mechanism of insect odorant receptors.

In this study, we investigated the pore dynamics of *Agam*Orco homotetramers by performing MD simulations on TBM and AF3 *Agam*Orco structural models, with bound agonist and antagonist, and using the complete *Ab*Orco model as reference. All *apo* and antagonist-bound models show the pore in a closed inactive state from consensus replicate cMD studies. The TBM model alone is able to effectively capture pore opening upon agonist binding, concordant with experimental studies (Tsitoura *et al*. 2015), comparable to pore opening in *Ab*Orco, while the AF3 HT model remains closed. The open pore state was confirmed by extending the cMD simulations, followed by GaMD simulations. Metadynamics is another enhanced sampling technique used to overcome energy barriers in MD simulations, but differs fundamentally from GaMD and requires predefined ‘collective variables’ to selectively push a system out of energy minima (Bhattarai and Miao 2018). On the other hand, GaMD uses an unguided, system-wide harmonic boost potential, and provides an unbiased enhanced sampling approach. We therefore used GaMD to further evaluate the conformational stability of the agonist-bound TBM *Agam*Orco HT after the pore-opening transition observed during cMD simulations. GaMD investigated whether the open pore conformation was retained and characterized the asymmetric nature of the ligand-induced pore-opening transition.

In the *apo* state, both TBM and AF3 *Agam*Orco models maintained stable, constricted pore architectures throughout the 500 ns cMD simulations, with no substantial increase in inter-chain gate distances. RMSD, pore-RMSD, RMSF, and Rg analyses showed that the TBM model closely resembled that of the *apo Ab*Orco template with moderate local pore fluctuation, whereas the *apo* AF3 model showed higher pore RMSD, but lower pore RMSF, likely reflecting the overall pore conformational changes rather than increased residue level fluctuation. Also, the minimum gate-region pore radius remained below 1 Å in both, and visualization confirmed that the pore architecture remained closed and non-conductive, consistent with the *apo Ab*Orco HT model.

VUAA1 binding produced distinct structural responses in the TBM *Agam*Orco model, where the analysis of binding interactions identified all five key binding-pocket residues and binding energy analysis supported stable, persistent agonist interactions within the orthosteric pocket. In contrast, the AF3 model captured only three key residues, suggesting that it adopted a slightly different ligand-binding conformation. In the TBM VUAA1-bound complex, inter-chain gate distance analysis showed that asymmetric pore widening began at 100 ns, mainly along the AC axis, while the BD axis remained stable or slightly contracted. This asymmetric open conformation persisted throughout the 1 µs simulation and was further extended using GaMD, which sampled larger pore radii and broader inter-chain distances, particularly around Gln476 and Leu477. The TBM VUAA1-bound model maintained a globally stable but locally flexible open-pore conformation associated with agonist-induced pore expansion. RMSD, pore-RMSD, RMSF, and Rg analyses were consistent with these findings, showing global conformational changes, increased pore-region deviation, increased local fluctuations around Val475, Gln476, and Leu477, and preserved global compactness without structural destabilization. Also, the agonist promoted increased conformational flexibility, driven by helix reorientation rather than large-scale helix tilting, accompanied by reorganization of *S7b* stabilizing interactions. Moreover, our results were consistent with the *Ab*Orco template model in that the TBM *Agam*Orco model reproduced key features expected for agonist-induced activation, including asymmetric inter-chain distance changes in gate residues consistent with pore opening, increased pore-region deviation, and preserved global structural stability, whereas the AF3 model did not undergo the agonist-associated pore expansion as observed in the TBM model. It should be noted that enhanced sampling simulations were performed only for the TBM agonist-bound system, as cMD simulations captured a clear pore-opening transition within the initial simulation timeframe, consistent with the template model, allowing GaMD to further investigate the structural characteristics and asymmetric nature of this pore-opening transition. In contrast, the agonist-bound AF3 system remained in a closed-like conformation during cMD simulations and did not exhibit an identifiable pore-opening transition within the simulated timeframe.

Both TBM and AF3 models showed close structural similarity to the *Ab*Orco template, indicating preservation of the overall tetrameric fold. Furthermore, the initial gate distances between opposing chain pairs (AC and BD) were comparable between the two models and closely resembled the *Ab*Orco template, suggesting similar starting pore geometries, despite differences in local conformational features. However, differences in their ligand binding pocket environments may contribute to their distinct activation responses. Docking analysis revealed differences in the VUAA1-binding pocket. Two of the five key residues, Phe81 and Phe82, involved in VUAA1 binding pocket in the TBM model and consistent with the experimental findings, were not captured in the AF3 model, resulting in different protein–ligand interaction patterns. These differences may influence the ability of VUAA1 to induce the conformational rearrangements required for *S7b* helix movement and pore opening. Therefore, the distinct activation behaviours observed between the TBM and AF3 models may be associated with differences in local binding-site architecture rather than differences in the overall tetrameric fold.

IPC occupied the same orthosteric binding pocket as VUAA1 in both TBM- and AF3-derived *Agam*Orco models, but its binding was weaker than that of VUAA1. Although IPC displayed less favourable GB and PB energy values than VUAA1, the difference may partly arise from its smaller size and fewer interactions and therefore does not necessarily reflect a proportional difference in binding affinity. Inter-chain gate distances, structural stability and flexibility analyses, and the pore radial profile analysis showed that IPC did not induce substantial pore opening in either model. Also, IPC binding maintains a more stable *S7b* conformation with limited helix rearrangement, supporting stabilization of a closed or non-functional channel state. Overall, IPC likely blocks agonist binding while maintaining a closed, non-conductive pore conformation. In the VUAA1-bound complexes, the conserved residues Ser144 and Tyr392 were observed in most interaction profiles, suggesting their critical role in agonist stabilization in both models. Similarly, IPC-bound TBM and AF3 complexes also showed stable interactions across all four chains, with Ser144 and Tyr392 frequently involved in ligand stabilization, which suggest that both VUAA1 and IPC remain stably bound within the orthosteric ligand-binding pocket during the simulations. The agonist VUAA1 binds strongly to *Agam*Orco, likely contributing to the receptor’s functional activity, correlating with the experimental EC50 value (58.5 μM) for the very similar OrcoRAM2 (Tsitoura *et al.* 2015), compared to IPC, with experimental IC50 of 41.7 μΜ (Kythreoti *et al.* 2024). The sustained favourable binding suggests that VUAA1 forms deep and stable interactions within the binding pocket, which may help maintain the receptor in an active state and facilitate the molecular mechanisms needed for pore opening. Although AF3 VUAA1-bound complexes exhibited favourable binding energies, these were slightly lower than those observed in the TBM model, the closed pore state suggests a non-functional structural model. In contrast, IPC binding was energetically more favourable in the TBM model than in AF3, although its binding energies were weaker than those observed for VUAA1. This suggests that IPC forms relatively stable but weaker interactions with *Agam*Orco, consistent with its role in maintaining a closed, inactive pore state.

Furthermore, antagonist-displacement simulation analysis showed that IPC binding shifts the agonist-induced open pore towards a more constricted closed pore conformation, confirmed by both cMD and GaMD simulations. The ligand occupancy simulations with different VUAA1 to IPC ratios provided insights into competitive modulation of Orco channel activity. The 3:1 VUAA1 to IPC occupancy condition resulted in pore closure, suggesting that low antagonist occupancy may influence channel gating even in the presence of bound agonist molecules and that low antagonist concentrations are sufficient to repel disease-causing insect pests. These findings provide experimentally testable hypotheses regarding ligand stoichiometry and regulation of Orco activity. More broadly, this study highlights the potential of computational approaches to identify ligand combinations and stoichiometric effects that modulate receptor activity, thereby guiding experimental investigations by prioritizing specific ligand ratios and receptor states for functional assays and strengthening the integration of computational modelling with experimental approaches in the development of novel vector-control strategies.

AF3 and TBM are both valuable structural modelling approaches. In fact, AF3 provides accurate models for sequences which have no known structural homologue. However, in the case of insect odorant receptors, despite predicting the fold accurately, the AF3 model was unable to support the functional pore opening by agonist, which is attributable to minor changes in the binding pocket residue interactions. We noted this deficiency in our previous studies on iOR monomers (Chidambara Thanu *et al.* 2024, Jabeen *et al.* 2024, Chidambara Thanu *et al.* 2025). Despite working well for predicting biomolecular interactions (Abramson *et al.* 2024), the *Agam*Orco AF3 HT model could not reproduce experimental ligand gating and pore opening observations. The AF3 model is possibly trapped in a conformational collapse to a single dominant conformation as noted recently (Maddipatla *et al.* 2026).

Thus, TBM appears to be better suited for iOR structural modelling at the present time. The TBM model presented in this study captured the structural features required for agonist-induced pore opening and ligand-mediated conformational transitions of *Agam*Orco HT. This highlights the importance of benchmarking predicted structures against functional criteria for specific biological applications. Furthermore, AF3 continues to evolve, and future improvements may enable more accurate prediction of functionally relevant conformational states.

Since there is no experimental Orco HT structure with bound agonist or antagonist, the TBM model developed in this study serves as an *in silico* system for structure-based discovery of *Agam*Orco antagonists. Beyond *A. gambiae*, the TBM pipeline presented in this study provides a structure-based framework for investigating Orco function across other disease-vector species. The integration of structure prediction combined with MD simulations can facilitate the analysis of ligand interactions, conformational changes, and gating mechanisms in less-characterized vector species. Such insights will support the future development and screening of novel Orco-targeting agonists or antagonists, with potential applications for novel insect attractants, repellents and vector-control strategies.

## Supplementary Material

vbag295_Supplementary_Data

## Data Availability

The data described in this article are provided in the Supplementary materials.
