# Supplementary material for: Decoding channel pore dynamics mechanism in Anopheles gambiae Orco homotetramer
Source: Bioinform Adv. 2026 Oct 6;6(1):vbag295. doi: 10.1093/bioadv/vbag295 (PMC13679930; doi:10.1093/bioadv/vbag295)
Supplement: Supplementary file 1 [file vbag295_supplementary_data.pdf]

Decoding Channel Pore Dynamics Mechanism in *Anopheles gambiae* Orco Homotetramer

Vaanathi Chidambara Thanu, Amara Jabeen, Georgia Kythreoti, Kostas Iatrou, Spyros E. Zographos, Philip W. Taylor and Shoba Ranganathan

Supporting Information

List of Figures:

Figure S1. Ligands used in this study 4

Figure S2. Comparison of *Agam*Orco homotetramer models with the experimental template *Ab*Orco (PDB ID: 6C70) structure [Butterwick et al. 2018] 5

Figure S3. Sequence alignment of *Agam*Orco with *Ab*Orco (PDB ID: 6C70) generated by SWISS-MODEL 6

Figure S4. Channel pore of the initial *Agam*Orco models compared with the reference *Ab*Orco 7

Figure S5. 2D view of the VUAA1 binding pocket interactions in TBM *Agam*Orco model ..8

Figure S6. 2D view of the VUAA1 binding pocket interactions in AF3 *Agam*Orco model ...9

Figure S7. 2D view of the IPC binding pocket interactions in TBM *Agam*Orco model 10

Figure S8. 2D view of the IPC binding pocket interactions in AF3 *Agam*Orco model 11

Figure S9. Space-filling view of the channel pore after 500 ns cMD simulations 12

Figure S10. Principal component analysis (PCA) projection of TBM *Agam*Orco gate residue conformational states 16

Figure S11. RMSD profiles of the *Agam*Orco and *Ab*Orco homotetramer models over 500 ns of cMD simulations 17

Figure S12. Pore RMSD profiles of the *Agam*Orco and *Ab*Orco homotetramer models over 500 ns of cMD simulations 18

Figure S13. Pore RMSF profiles of the *Agam*Orco and *Ab*Orco homotetramer models over 500 ns of cMD simulations 19

Figure S14. Radius of gyration profiles of the *Agam*Orco and *Ab*Orco homotetramer models over 500 ns of cMD simulations 20

Figure S15. Analysis of *Agam*Orco complex with agonist VUAA1 in GaMD simulations....21

Figure S16. Inter-chain distance measurement of pore-gate residue Gln476 in *Agam*Orco homotetramer models post simulation. 22

Figure S17. Interaction of VUAA1 in TBM *Agam*Orco model after 500 ns simulation 23

Figure S18. Interaction of VUAA1 in AF3 *Agam*Orco model after 500 ns simulation 24

Figure S19. Interaction of IPC in TBM *Agam*Orco model after 500 ns simulation 25
Figure S20. Interaction of IPC in AF3 *Agam*Orco model after 500 ns simulation 26
Figure S21. Open pore channel closes with antagonist binding in *Agam*Orco homotetramer 30
Figure S22. Post MD analysis of *Agam*Orco homotetramer simulations initiated from the cMD derived open pore conformation in *apo* and IPC-bound complex..33
Figure S23. Post MD analysis of *Agam*Orco homotetramer simulations initiated from GaMD derived open pore conformation in *apo* and IPC-bound complex 34
Figure S24. Post MD analysis of *Agam*Orco homotetramer simulations with different concentrations of VUAA1 and IPC 36

<u>List of Tables:</u>

Table S1. Initial inter-chain distances (Å) of gate residues in *Ab*Orco and *Agam*Orco models 7
Table S2. Distance analysis of pore-gate residues (mean inter-chain distances in Å) after 500 ns cMD simulations 13
Table S3. Distance analysis of pore-gate residues (mean inter-chain distances in Å) in GaMD simulations 14
Table S4. Helical RMSD differences between the initial (1 ns) and final (500 ns) conformations of TBM *Agam*Orco complexes (mean values from three independent replicates) 27
Table S5. Helix tilt and reorientation differences between the initial (1 ns) and final (500 ns) conformations of the *S7b* helices in TBM *Agam*Orco complexes (mean values from three replicates)..27
Table S6. Key changes in *S7b* hydrogen bond occupancy between the early (1-10 ns) and late (490-500 ns) phases of agonist-bound TBM *Agam*Orco complexes (mean values from three replicates)..28
Table S7. Energy analysis after 500 ns in VUAA1 and IPC binding in the TBM and AF3 *Agam*Orco model and VUAA1 binding in *Ab*Orco model 29
Table S8. Distance analysis (mean inter-chain distances in Å) after 200 ns and 500 ns of MD simulations of antagonist binding in the open pore conformations derived from cMD and GaMD 31
Table S9. Energy analysis after 500 ns simulation in IPC binding in the cMD and GaMD derived open pore conformation in TBM *Agam*Orco model. 34

Table S10. Inter-chain pore-gate distances (mean distances in Å) at different ligand concentrations after 500 ns simulation in TBM *Agam*Orco model 35
Table S11. Energy analysis after 500 ns in different ligand concentrations in TBM *Agam*Orco model..36

List of Notes:

Note S1. Membrane building and MD simulation details 4
Note S2. GaMD setup and protocol 4
Note S3. RMSD, RMSF and Rg analyses of *Ab*Orco and *Agam*Orco homotetramer models after cMD and post-GaMD simulations of TBM *Agam*Orco with VUAA1 14
Note S4. The open pore closes following antagonist binding 29
Note S5. RMSD, RMSF and Rg analyses for agonist derived open pore conformation and antagonist-bound in open conformation in TBM *Agam*Orco HT models 32
Note S6. The effect of agonist:antagonist concentration on pore dynamics in TBM *Agam*Orco model 35

References..37

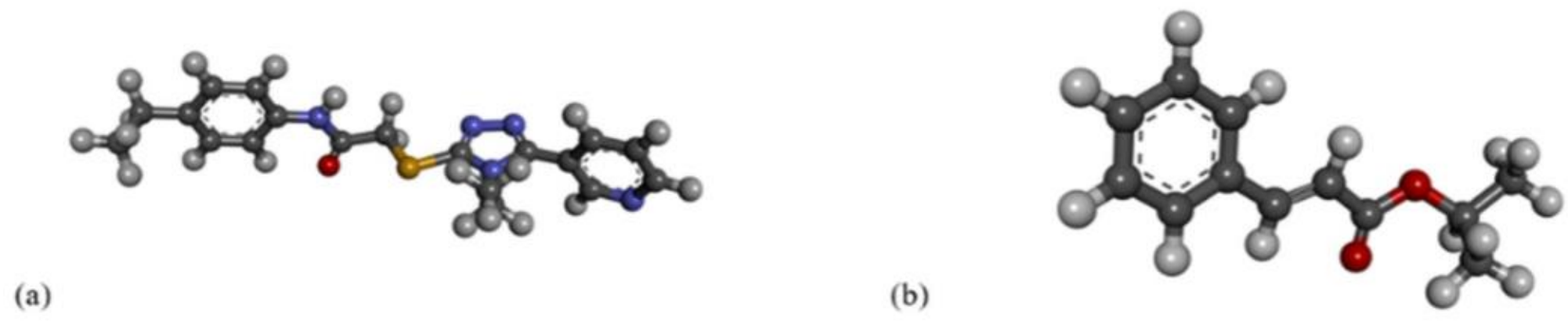

Figure S1. Ligands used in this study.

The chemical structure of the ligands used in this study (a) VUAA1 (CID:1319135), and (b) IPC (CID:5273464) in ball and stick format and with atom colours (C: grey, H: white, N: blue, O: red and S: yellow).

Note S1: Membrane building and MD simulation details

Simulations were carried out using Amber 22 [Case *et al.* 2022], with membrane systems constructed with CHARMM-GUI Membrane Builder [Wu *et al.* 2014] using the CHARMM-GUI server [Jo *et al.* 2008]. Membranes included POPC and POPE lipids in a 40:30 ratio, approximating the native composition of *A. gambiae* membranes [Moreno *et al.* 2021]. Previous simulation studies [Angladon *et al.* 2019, Yusuf *et al.* 2022] have included only these major lipid classes in membrane building, ignoring minor lipids as well as sterol molecules. The protein parameters were generated using the AMBER ff14SB force field [Maier *et al.* 2015]. Ligand parameters were generated using Antechamber with the General Amber Force Field (GAFF) [He *et al.* 2020]. The complexes were solvated with TIP3P water, and NaCl was added to a final ionic strength of 0.15 mM, with ion placement determined using the Monte Carlo method.

Given the large size and tetrameric architecture of the *Agam*Orco, a force constant of 5.0 kcal mol^{-1} $Å^{-2}$ was applied during the initial minimisation step (min1), followed by a reduced force constant of 2.0 kcal mol^{-1} $Å^{-2}$ in the second minimisation step (min2). During the heating phase, restraints were maintained at 2.0 kcal mol^{-1} $Å^{-2}$ and were subsequently reduced to 1.0 kcal mol^{-1} $Å^{-2}$ during the equilibration (hold) step to allow gradual relaxation of the protein. Simulations were run under periodic boundary conditions that defined an average simulation box volume for each. Equilibration was performed under the NPT ensemble at constant pressure with a temperature set to 298.15 K, using the Nose–Hoover thermostat, approximating physiological conditions in insects. Conventional MD (cMD) production simulations were conducted under the NVE ensemble, with no pressure control or barostat applied.

A minimum of three independent replicates of 500 ns were performed for each HT model, with additional replicates where necessary to achieve concordant results.

Note S2: GaMD setup and protocol

GaMD simulations [Wang *et al.* 2021] were performed using an open pore structure selected from the equilibrated cMD trajectory between 900 ns and 1 µs as the starting conformation. Weak harmonic distance restraints were applied between pore subunits at the hydrophobic gate to overcome the hydrophobic barrier, with restraint strengths applied in stages. The system was then relaxed without

restraints to allow the side chains to equilibrate in the open conformation. The equilibrated system was then used for GaMD production. To collect potential-energy statistics and determine GaMD boost parameters, an initial 10 ns GaMD equilibration run was carried out, followed by GaMD production simulations under constant temperature and pressure conditions. GaMD simulations were performed using AMBER 22 [Case *et al.* 2022], with dual-boost GaMD (igamd = 3), boosting both dihedral and total potential. Production was run in consecutive blocks (Prod-1, Prod-2), each 100 ns in length, without distance restraints.

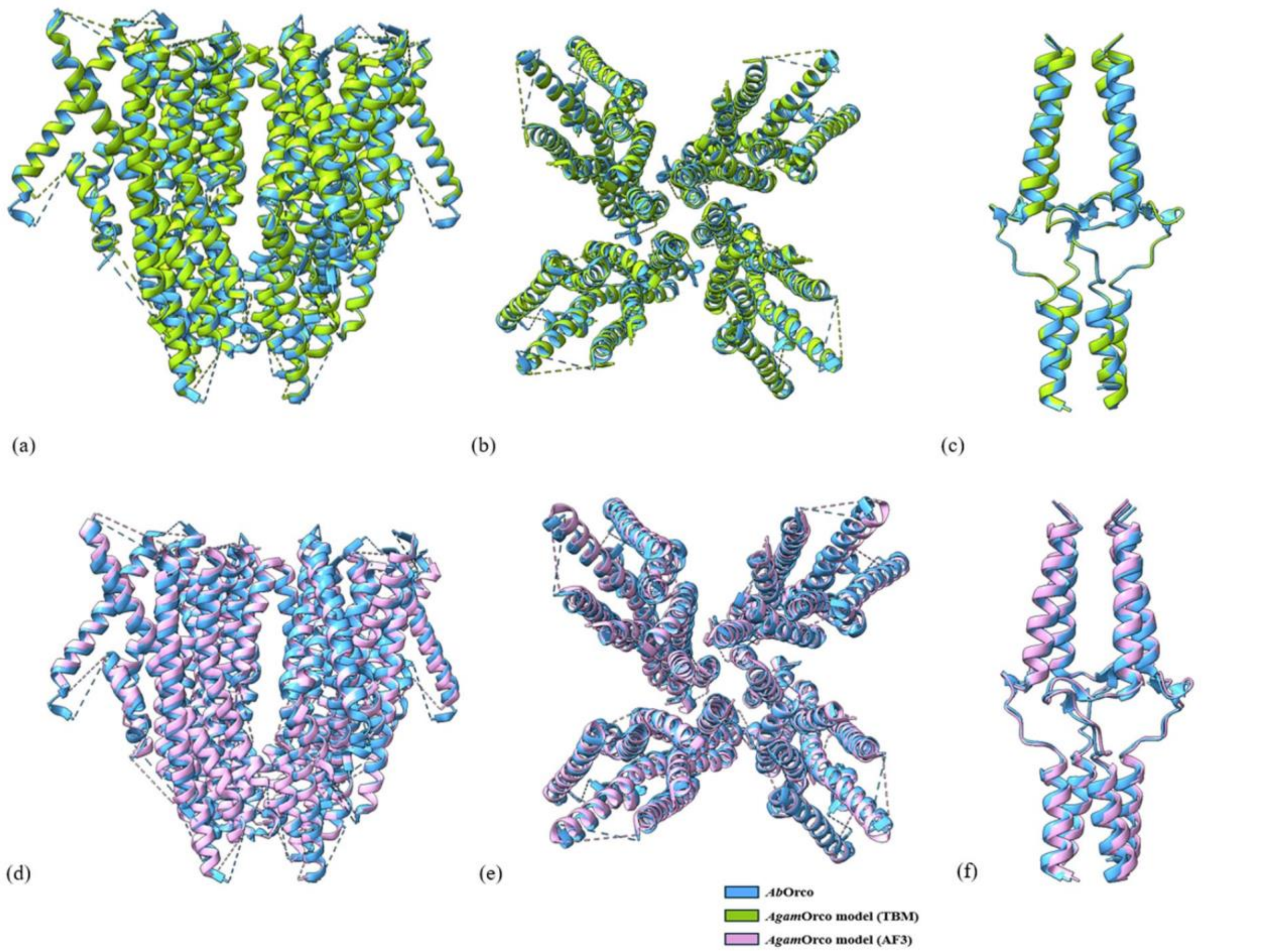

Figure S2. Comparison of *Agam*Orco homotetramer models with the experimental template *Ab*Orco (PDB ID: 6C70) structure [Butterwick *et al.* 2018].

Superimposition of TBM model of *Agam*Orco with the experimental structure *Ab*Orco (PDB ID: 6C70) showing only the helices: (a) side view, (b) the top view, and (c) pore region. Superimposition of AF3 model of *Agam*Orco with the experimental structure *Ab*Orco, showing only the helices in (d) side view, (e) the top view, and (f) pore region.

```
                                                S2
6C70_AbOrco          VNLMMESDDVDDLTANTITMLFFLHPIVKMIYFPVRSKIFYKTLAIWNNPNSHPLFAESN

Q7QCC7_AgamOrco      GNLATNADDVNELTANTIITLFFTHSVTKFIYFAVNSENFYRTLAIWNQTNTHPLFAESD

                      **    ***  ******  *** *   * *** * *  ** ******  * *******

                                  S3
6C70_AbOrco          AREHALAITKMRRLLFCVAGATIFSVISWTGITFIEDSvkritdpetnettiiPIPRLMI

Q7QCC7_AgamOrco      ARYHSIALAKMRKLLVLVMATTVLSVVAWVTITFFGESVKTVLDKATNETYTVDIPRLPI

                     ** *  *  *** **  *   *  **  *  ***                    **** *

                                                   S4
6C70_AbOrco          RTFYPFNAMSGAGHVFALIYQFYYLVISMAVSNSLDVLFCSWLLFACEQLQHLKAIMKPL

Q7QCC7_AgamOrco      KSWYPWNAMSGPAYIFSFIYQIYFLLFSMVQSNLADVMFCSWLLLACEQLQHLKGIMRSL

                        ** *****    *  *** * *  **  **  ** ****** ********* **  *


6C70_AbOrco          MELSATldtvvpnsgelfkagsadhlresqgvqpsgngdnvldvdlrgivsnrqdfta--

Q7QCC7_AgamOrco      MELSASLDTYRPNSSQLFRAISAGSKSELIINEEKDPDVKDFDLSGIYSSKADWGAQFRA

                     *****

                                                             S5
6C70_AbOrco          tfrptagttfnggvgpnGLTKKQEMLVRSAIKYWVERHKHVVRLVTAVGDAYGVALLLHM

Q7QCC7_AgamOrco      PSTLQTFDENGRNGNPNGLTRKQEMMVRSAIKYWVERHKHVVRLVSAIGDTYGPALLLHM

                                        * **** ******************* * ** ** ******

                                                          S6
6C70_AbOrco          LTTTITLTLLAYQATKVNGVNVYAATVIGYLLYTLGQVFLFCIFGNRLIEESSSVMEAAY

Q7QCC7_AgamOrco      LTSTIKLTLLAYQATKIDGVNVYGLTVIGYLCYALAQVFLFCIFGNRLIEESSSVMEAAY

                     ** ** **********  *****  ****** * * ************************

                                S7a                            S7b
6C70_AbOrco          SCHWYDGSEEAKTFVQIVCQQCQKAMSISGAKFFTVSLDLFASVLGAVVTYFMVLVQLK

Q7QCC7_AgamOrco      SCHWYDGSEEAKTFVQIVCQQCQKAMTISGAKFFTVSLDLFASVLGAVVTYFMVLVQLK

                     ************************** ********************************
```

Figure S3. Sequence alignment of *Agam*Orco with *Ab*Orco (PDB ID: 6C70) generated by SWISS-MODEL.

The helices (*S1–S7*) are marked, and missing residues in the *Ab*Orco PDB structure are in white font against a black background, using the complete *Ab*Orco Uniprot sequence, B0FAQ4.

Table S1: Initial inter-chain distances (Å) of gate residues in *Ab*Orco and *Agam*Orco models.

Initial	*Apo Ab*Orco		Initial	*Apo* TBM *Agam*Orco		*Apo* AF3 *Agam*Orco	
	A-C	B-D		A-C	B-D	A-C	B-D
Val469	12.35	12.52	Val473	12.32	12.16	12.29	12.37
Leu470	16.30	16.46	Leu474	16.38	16.23	16.48	16.63
Val471	20.24	20.42	Val475	20.51	20.34	20.33	20.44
Gln472	15.12	15.29	Gln476	15.17	15.00	15.05	15.12
Leu473	11.25	11.07	Leu477	11.30	11.00	10.42	10.56

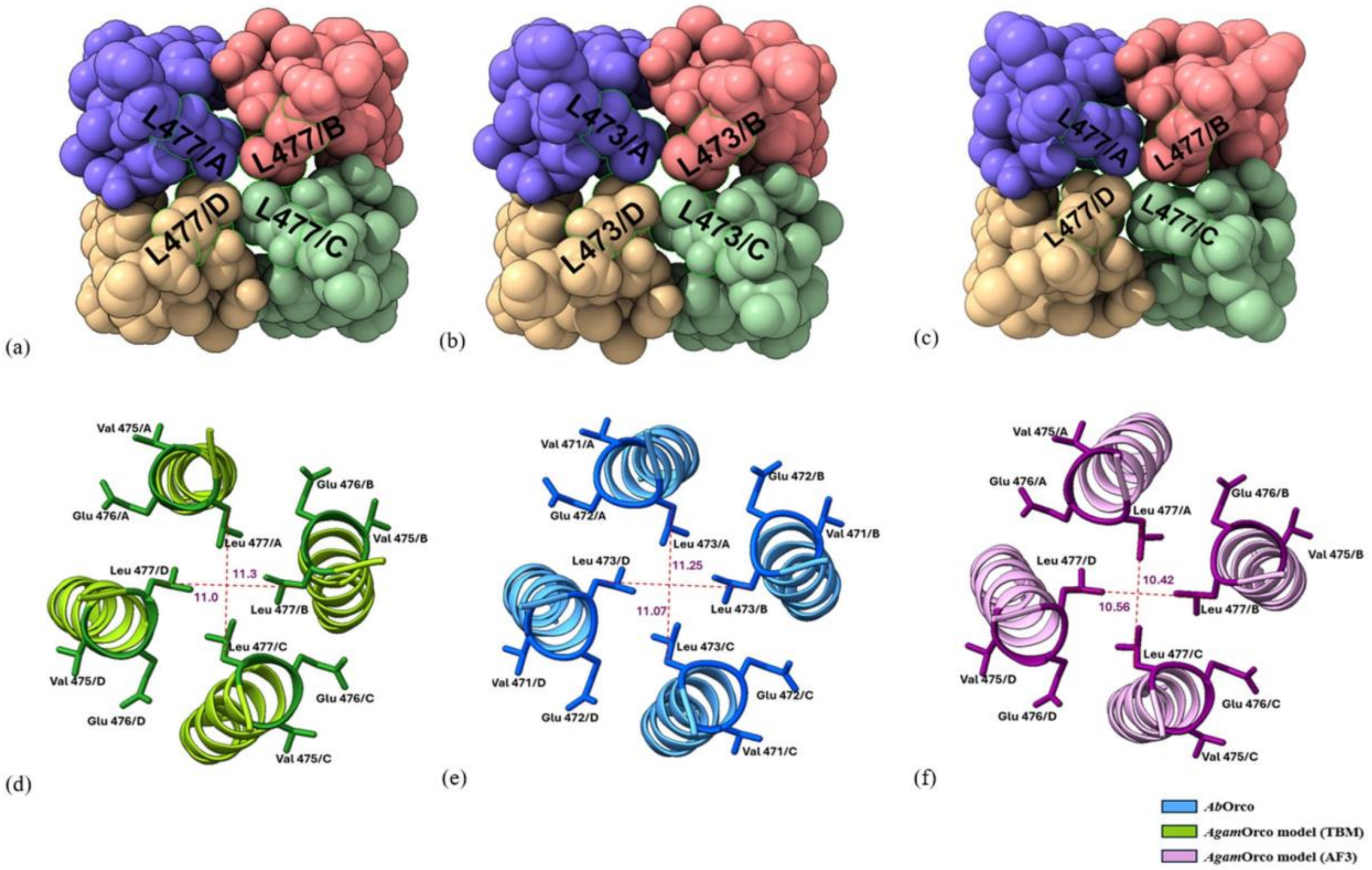

Figure S4. Channel pore of the initial *Agam*Orco models compared with the reference *Ab*Orco.

Top view of the pore in (a) *Ab*Orco, (b) *Agam*Orco model generated using TBM, (c) *Agam*Orco model generated using AF3, (d) Inter-chain distance of the residue Leu477 between chain pairs A-C and B-D in the TBM generated *Agam*Orco model, (e) Inter-chain distance of the residue Leu473 between chain pairs AC and BD in the reference *Ab*Orco model, (f) Inter-chain distance of the residue Leu477 between chain pairs AC and BD in the AF3 generated *Agam*Orco model.

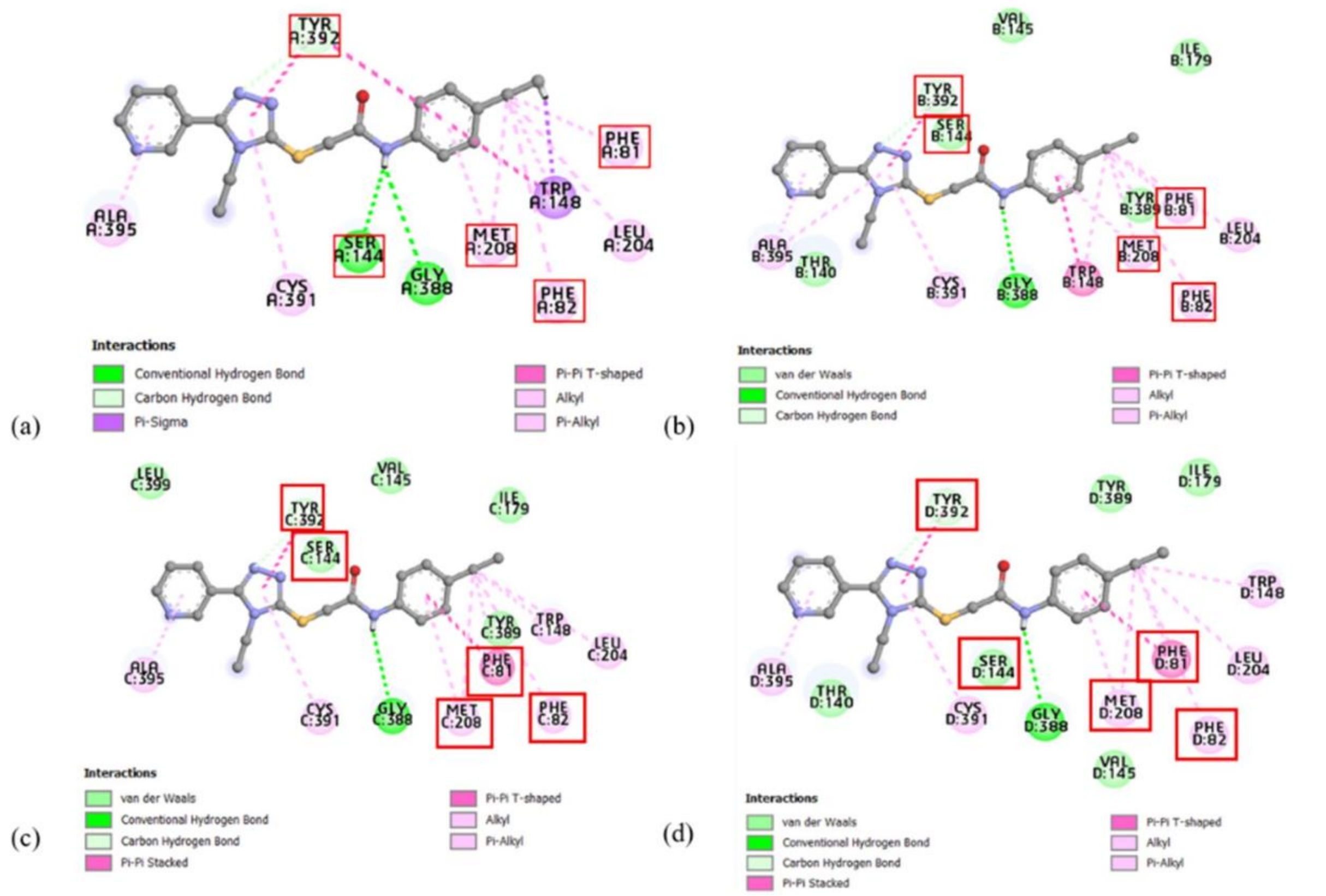

Figure S5. 2D view of the VUAA1 binding pocket interactions in TBM *Agam*Orco model.

Interacting residues of VUAA1 are shown for chains (a) A, (b) B, (c) C and (d) D of the TBM *Agam*Orco model. The conserved *Dmel*Orco binding pocket residues as reported [Pacalon *et al*. 2023] are boxed in red.

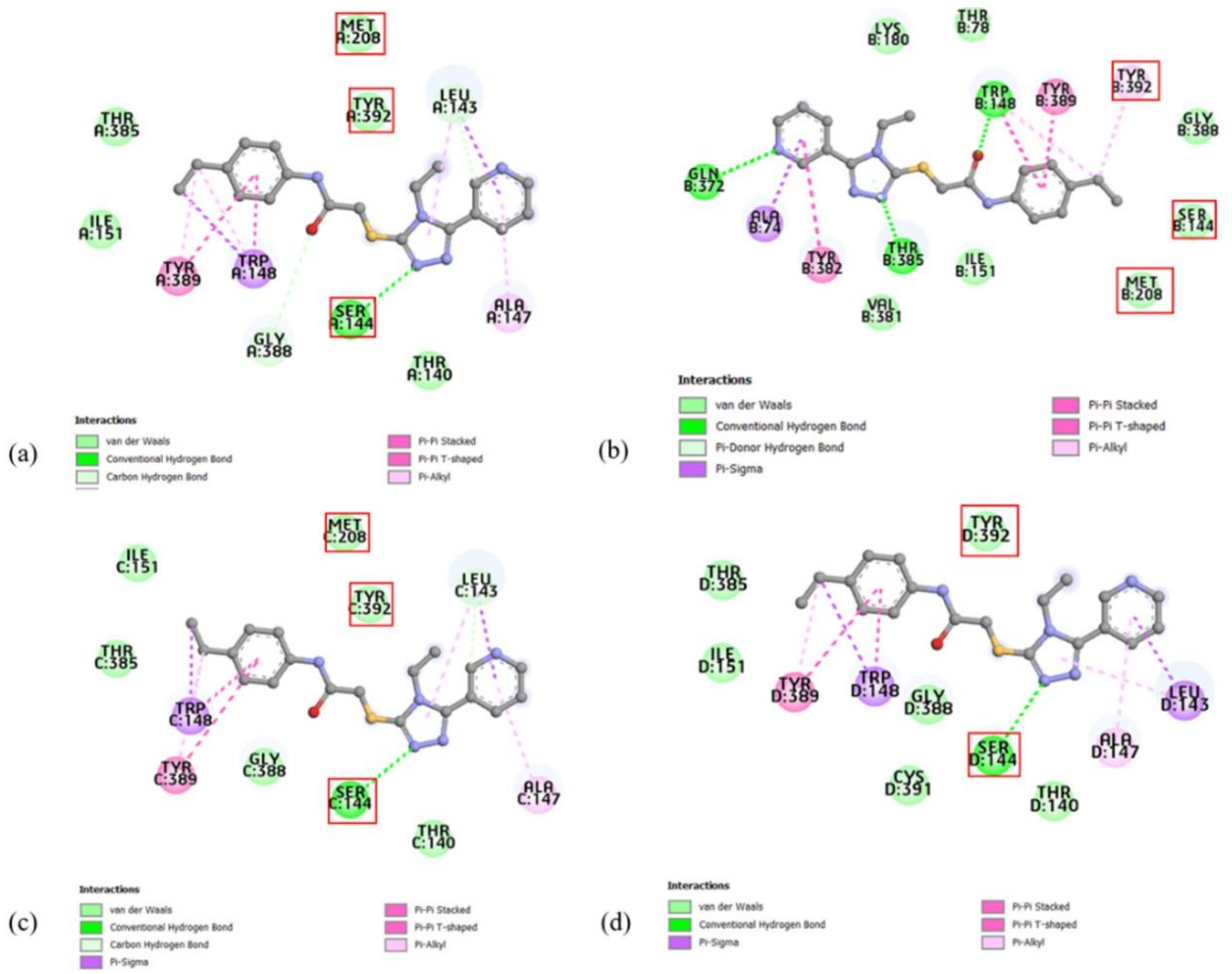

Figure S6. 2D view of the VUAA1 binding pocket interactions in AF3 *Agam*Orco model.

Interacting residues of VUAA1 are shown for chains (a) A, (b) B, (c) C and (d) D of the AF3 *Agam*Orco model. The conserved *Dmel*Orco binding pocket residues as reported [Pacalon *et al*. 2023] are boxed in red.

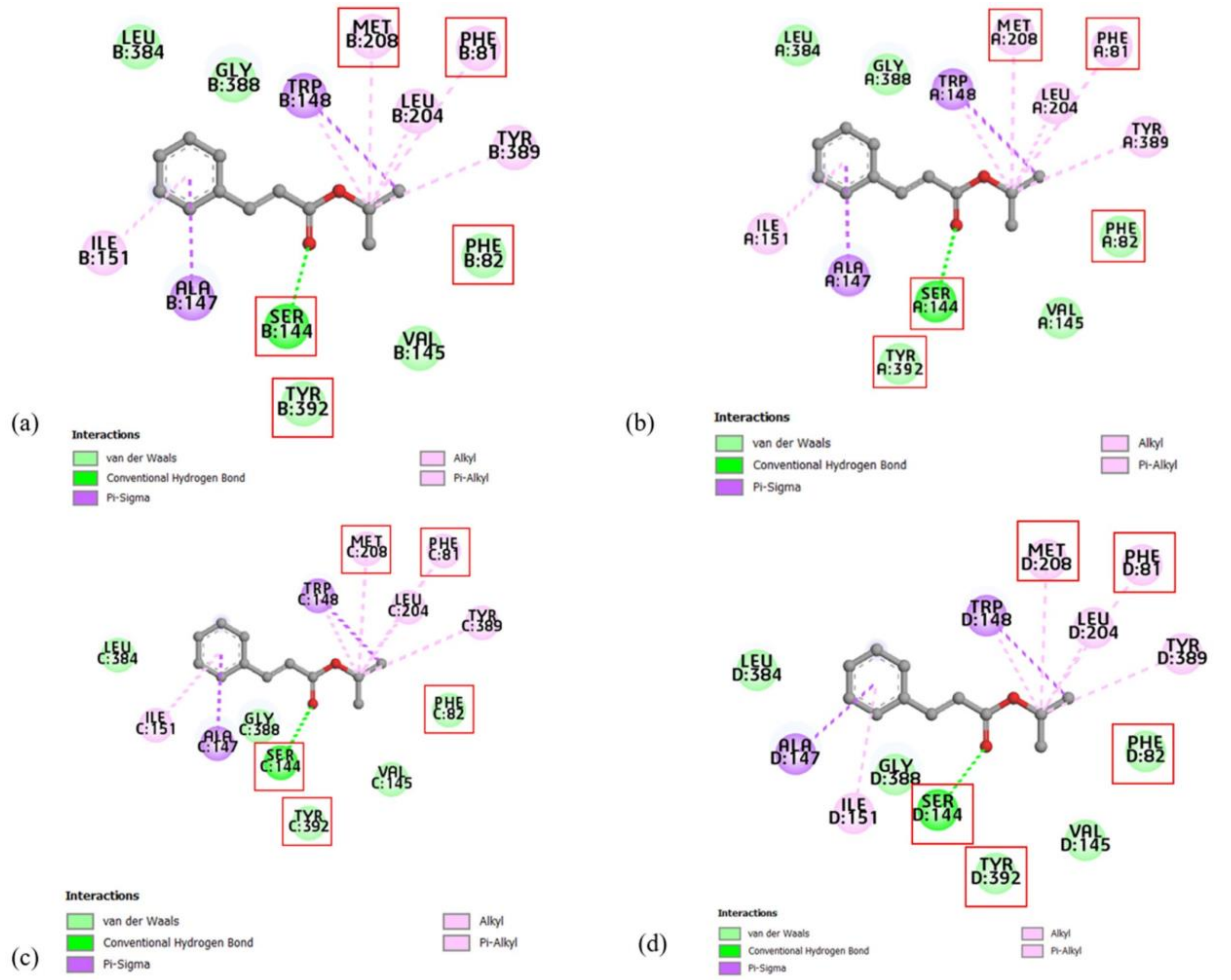

Figure S7. 2D view of the IPC binding pocket interactions in TBM *Agam*Orco model.

Interacting residues of IPC are shown for chains (a) A, (b) B, (c) C and (d) D of the TBM *Agam*Orco model. The conserved *Dmel*Orco binding pocket residues as reported [Pacalon *et al.* 2023] are boxed in red.

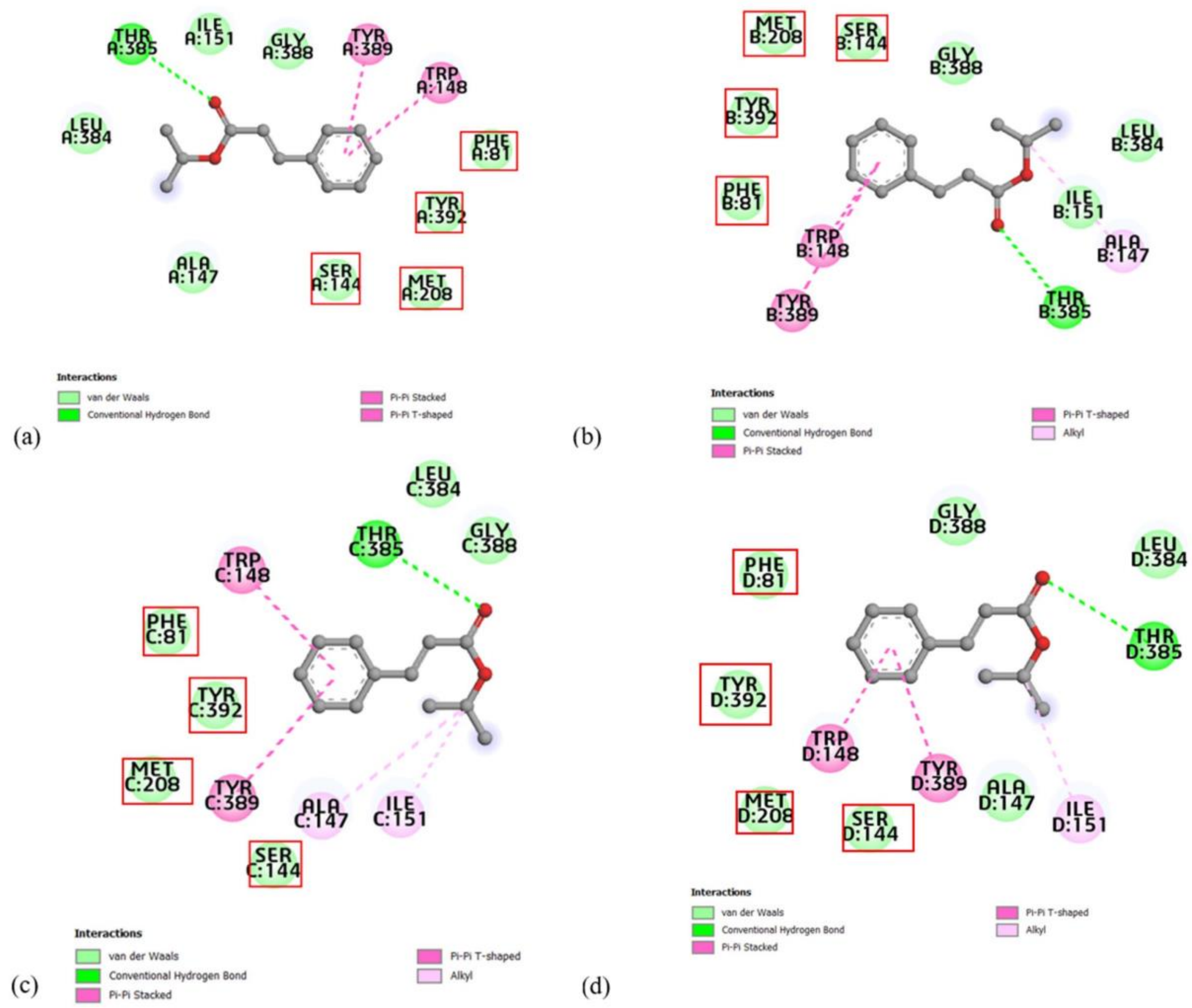

Figure S8. 2D view of the IPC binding pocket interactions in AF3 *Agam*Orco model.

Interacting residues of IPC are shown for chains (a) A, (b) B, (c) C and (d) D of the AF3 *Agam*Orco model. The conserved *Dmel*Orco binding pocket residues as reported [Pacalon *et al.* 2023] are boxed in red.

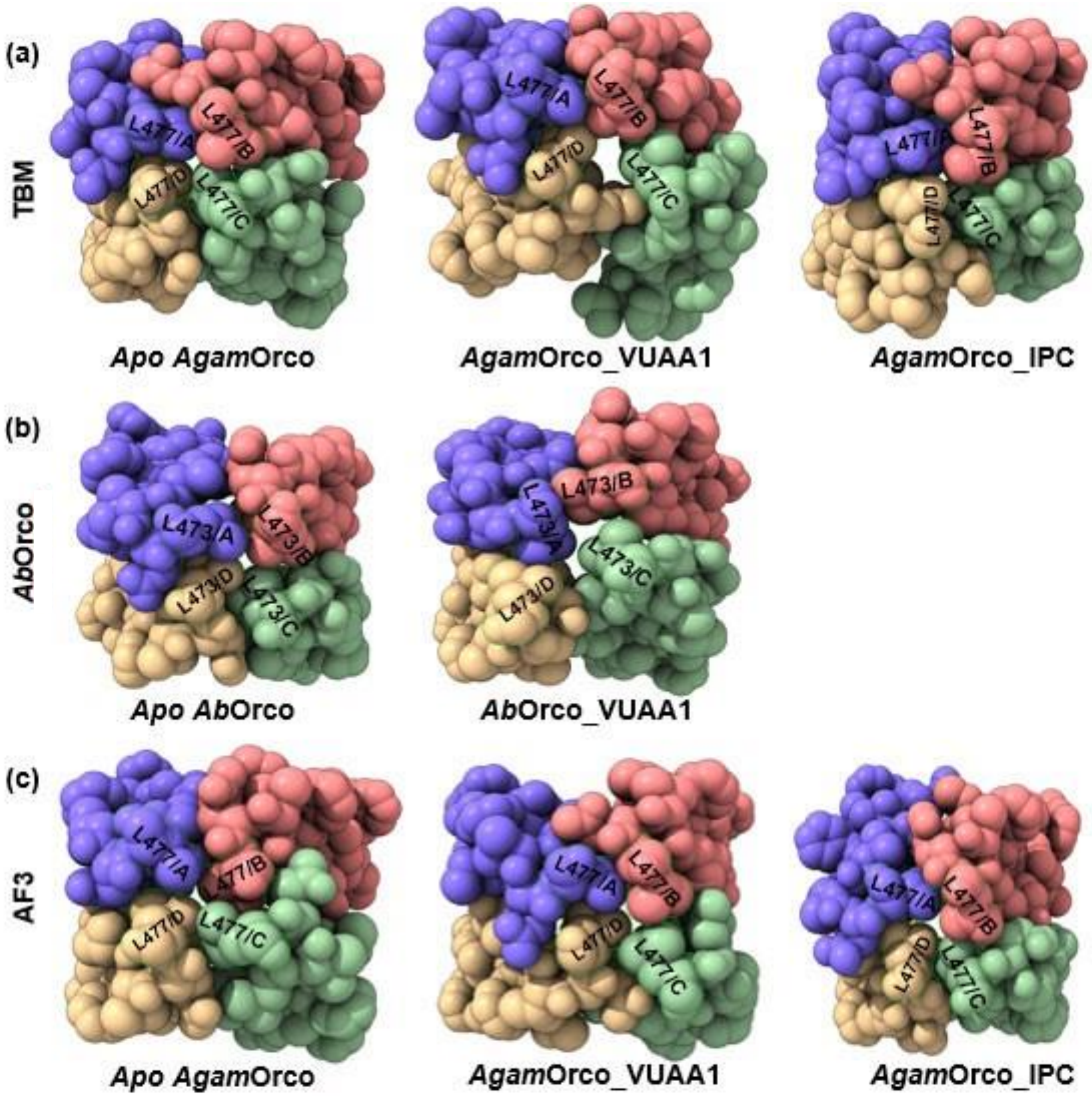

Figure S9. Space-filling view of the channel pore after 500 ns cMD simulations.

(a) *apo*, VUAA1-bound, and IPC-bound models of TBM *Agam*Orco, (b) *apo* and VUAA1-bound models of *Ab*Orco, and (c) *apo*, VUAA1-bound, and IPC-bound models of AF3 *Agam*Orco. The gating residue L477 in *Agam*Orco and L473 in *Ab*Orco are labelled.

Table S2. Distance analysis of pore-gate residues (mean inter-chain distances in Å) after 500 ns cMD simulations.

Apo	***Ab*Orco**		***Apo***	**TBM *Agam*Orco**		**AF3 *Agam*Orco**	
	**A-C**	**B-D**		**A-C**	**B-D**	**A-C**	**B-D**
Val469	12.61	10.70	Val473	12.13	12.33	12.69	10.99
Leu470	16.40	13.37	Leu474	14.25	15.33	16.21	13.33
Val471	20.90	18.18	Val475	19.52	19.70	21.03	18.90
Gln472	15.90	14.10	Gln476	15.13	14.82	16.07	14.82
Leu473	11.83	9.60	Leu477	9.01	9.78	11.35	9.92
**With VUAA1**	***Ab*Orco**		**With VUAA1**	**TBM *Agam*Orco**		**AF3 *Agam*Orco**	
	**A-C**	**B-D**		**A-C**	**B-D**	**A-C**	**B-D**
Val469	10.18	13.92	Val473	15.48	9.48	11.59	12.24
Leu470	13.85	17.27	Leu474	18.25	14.15	14.82	15.84
Val471	18.09	22.51	Val475	23.21	18.55	19.63	20.32
Gln472	13.12	18.33	Gln476	18.77	14.25	15.26	15.79
Leu473	8.51	13.33	Leu477	13.05	10.36	10.22	10.92
**With IPC**	***Ab*Orco**		**With IPC**	**TBM *Agam*Orco**		**AF3 *Agam*Orco**	
	**A-C**	**B-D**		**A-C**	**B-D**	**A-C**	**B-D**
Val469	NA	NA	Val473	10.21	12.95	12.92	10.10
Leu470	NA	NA	Leu474	13.50	16.38	16.68	12.70
Val471	NA	NA	Val475	18.60	21.05	21.44	17.87
Gln472	NA	NA	Gln476	14.34	16.31	16.55	13.91
Leu473	NA	NA	Leu477	8.98	11.15	12.09	9.35

Table S3. Distance analysis of pore-gate residues (mean inter-chain distances in Å) in GaMD simulations.

Distance analysis in 100- 200 ns GaMD simulations in VUAA1 bound TBM *Agam*Orco		
**Residue**	**A-C**	**B-D**
Val473	15.85±0.47	8.81±0.38
Leu474	18.31±0.41	12.05±0.48
Val475	23.80±0.40	17.40±0.48
Gln476	19.89±0.49	16.06±0.63
Leu477	14.02±0.54	13.02±0.65

Note S3: RMSD, RMSF and Rg analyses of *Ab*Orco and *Agam*Orco homotetramer models after cMD and post-GaMD simulations of TBM *Agam*Orco with VUAA1

Analysing the RMSD profiles (Figure S11a) shows that the *apo* TBM *Agam*Orco model stabilised around approximately 5 Å and AF3 around approximately 4 Å, indicating overall structural stability. Both the models underwent early relaxation but subsequently maintained stable overall structures comparable to that of the *apo Ab*Orco reference model (Figure S11b). This shows that in both models, in the absence of ligand, the pore remains dynamically flexible but structurally constrained, consistent with a stable *apo* state conformation. Upon VUAA1 binding, TBM RMSD rose to approximately 5.6 Å till 350 ns and then increased to approximately 6 Å, which is maintained till 500 ns, showing agonist induced global rearrangement comparable to the *Ab*Orco reference model, while AF3-VUAA1 complex (Figure S11c) remained stable around approximately 4 Å, reflecting a compact and stable structure. IPC-bound systems stabilised around approximately 5.5-6 Å for TBM from 150 ns all the way to 500 ns and approximately 3.9-4 Å for AF3, indicating no significant conformational changes.

Analysing the pore RMSD profile (Figure S12a) showed that the TBM *apo* model remained low with deviations of around 0.75-1.5 Å, confirming stability of the pore region and comparable to the *apo Ab*Orco reference (Figure S12b). In contrast, the AF3 *apo* model (Figure S12c) showed larger pore RMSD fluctuations of around 1.5-2.5 Å, suggesting greater deviation of the pore region from its initial conformation. Upon VUAA1 binding, the TBM model showed an initial increase in pore RMSD to around 2-3 Å during the first 100 ns, followed by stabilisation around 1.5-2 Å, consistent with pore rearrangement towards an open conformation. However, the AF3-VUAA1 complex showed a stable pore RMSD around 1.5-2 Å, with only minor fluctuations, indicating that the pore region remains structurally stable and undergoes only small local conformational changes to accommodate the ligand. In the IPC-bound systems, pore RMSD remained low and stable in TBM (1.2-1.7 Å) and in AF3 (1.5-2.0 Å), after relaxation with only moderate fluctuations, indicating minimal structural deviation of the pore region confirming that IPC maintains a closed and structurally stable pore in both models.

The pore RMSF profiles (Figure S13a) showed that the *apo* TBM model exhibited moderate pore-gate residue flexibility, with Leu477 showing slightly higher fluctuation, while the AF3 *apo* model (Figure S13c), displayed relatively low RMSF values, closely comparable to the *apo AbOrco* reference (Figure S13b), indicating a more locally constrained pore-lining region. Upon binding VUAA1, the TBM model showed increased RMSF values, suggesting enhanced pore-region fluctuation associated with agonist induced conformational rearrangement. In contrast, the AF3-VUAA1 complex showed only minor fluctuations comparable to those of the reference model, with slightly increased fluctuations at Val473, Gln476, and Leu477, indicating limited pore-gate residue changes while maintaining overall pore stability.

The Rg plot showed that the *apo* TBM (Figure S14a) maintained stability comparable to the *apo AbOrco* (Figure S14b), while AF3 was slightly more compact (Figure S14c). VUAA1-bound TBM preserved overall structural stability, and AF3 remained compact. IPC-bound systems maintained global compactness, with TBM slightly higher than its *apo* and AF3 comparable to its *apo.* For the IPC-bound systems, the TBM complex showed uniformly low pore-residue fluctuations, including at the hydrophobic gate residues Val468 and Leu472, suggesting that IPC stabilises a dynamically restrained closed pore. AF3-IPC also showed generally low fluctuations, although Gln476 and Leu477 displayed slightly higher RMSF values of approximately 0.9-1.2 Å.

From the RMSD plot, GaMD simulations of TBM with bound VUAA1 (Figure S15a) stabilised around approximately 2 Å with very small fluctuations in the final 10 ns (SD 0.09 Å), indicating that the system remained stable during the production phase, supporting stable open pore conformations.

In the GaMD simulation (Figure S15b), RMSF of the pore-lining residues showed low to moderate fluctuations across the four chains, indicating that the open pore remained locally flexible but globally stable.

Post-GaMD Rg plot for the open pore conformation of the VUAA1-bound TBM (Figure S15c) showed that it maintained its overall compactness without large-scale expansion or collapse.

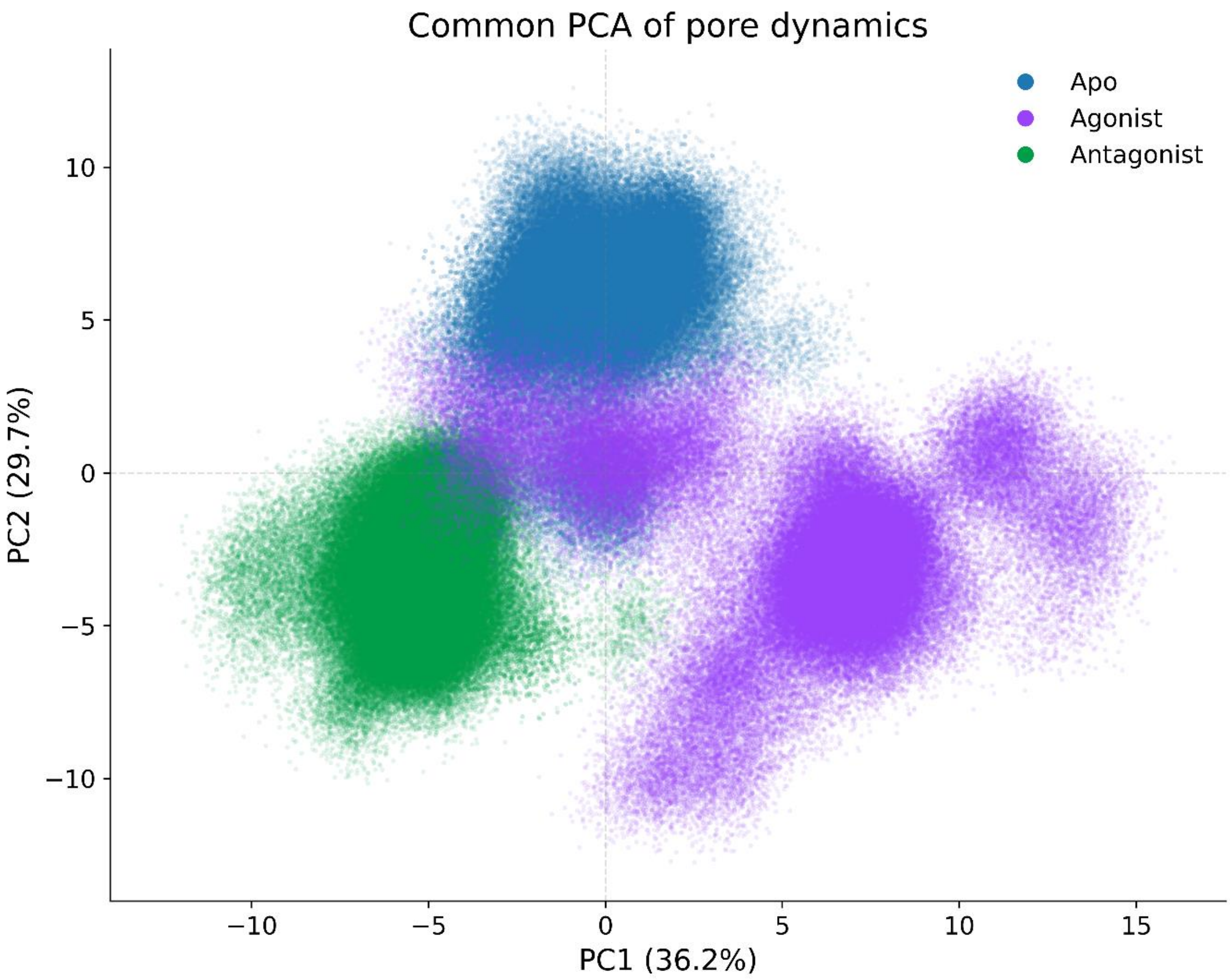

Figure S10. Principal component analysis (PCA) projection of TBM *Agam*Orco gate residue conformational states.

PCA projection of the gate residues in *apo*, VUAA1 (agonist) bound, and IPC (antagonist) bound TBM *Agam*Orco complexes using a common covariance matrix.

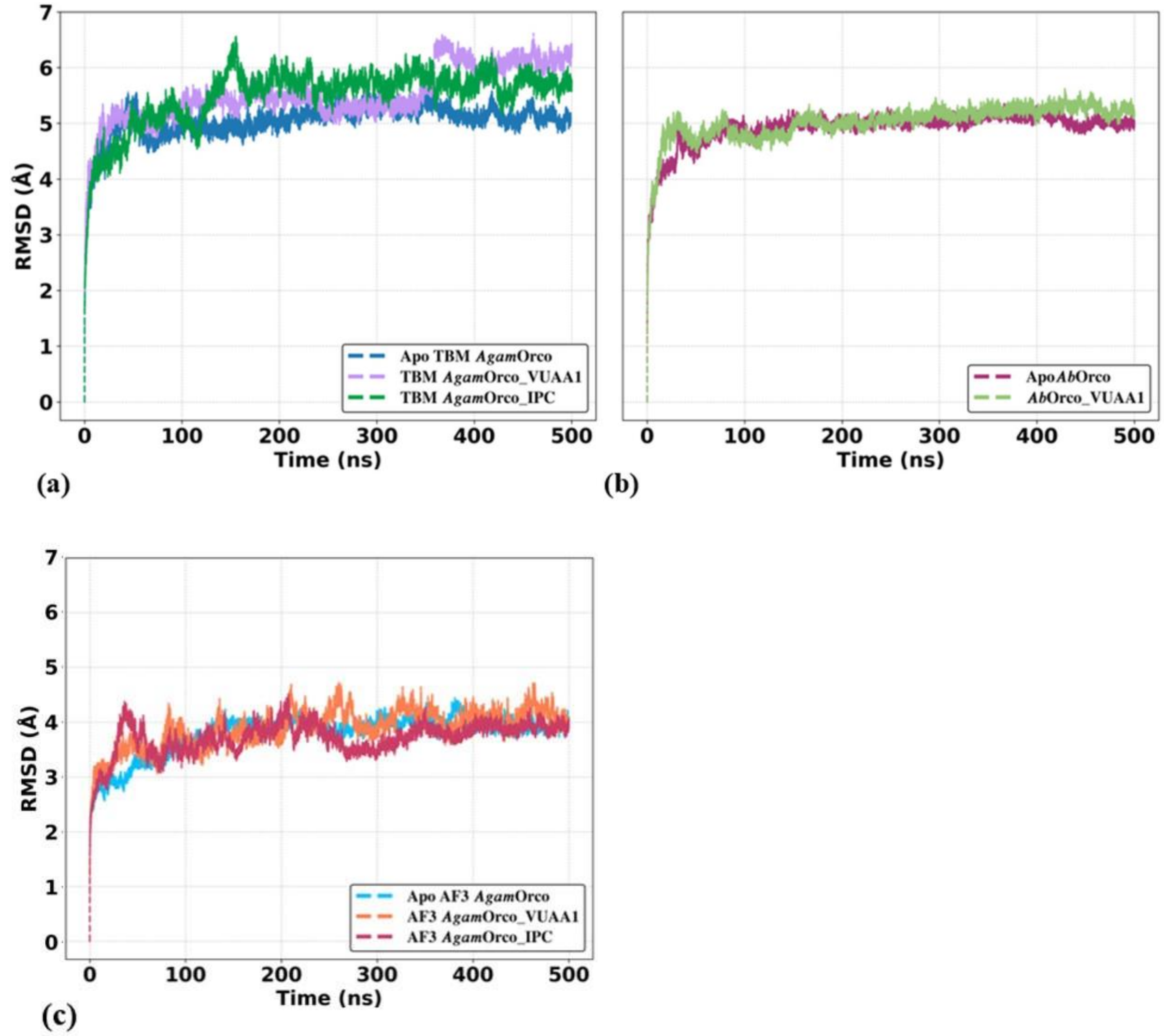

Figure S11. RMSD profiles of the *Agam*Orco and *Ab*Orco homotetramer models over 500 ns of cMD simulations.

(a) TBM *Agam*Orco in the *apo* state and in complex with VUAA1 or IPC, (b) *Ab*Orco in the *apo* state and in complex with VUAA1, (c) AF3 *Agam*Orco in the *apo* state and in complex with VUAA1 or IPC.

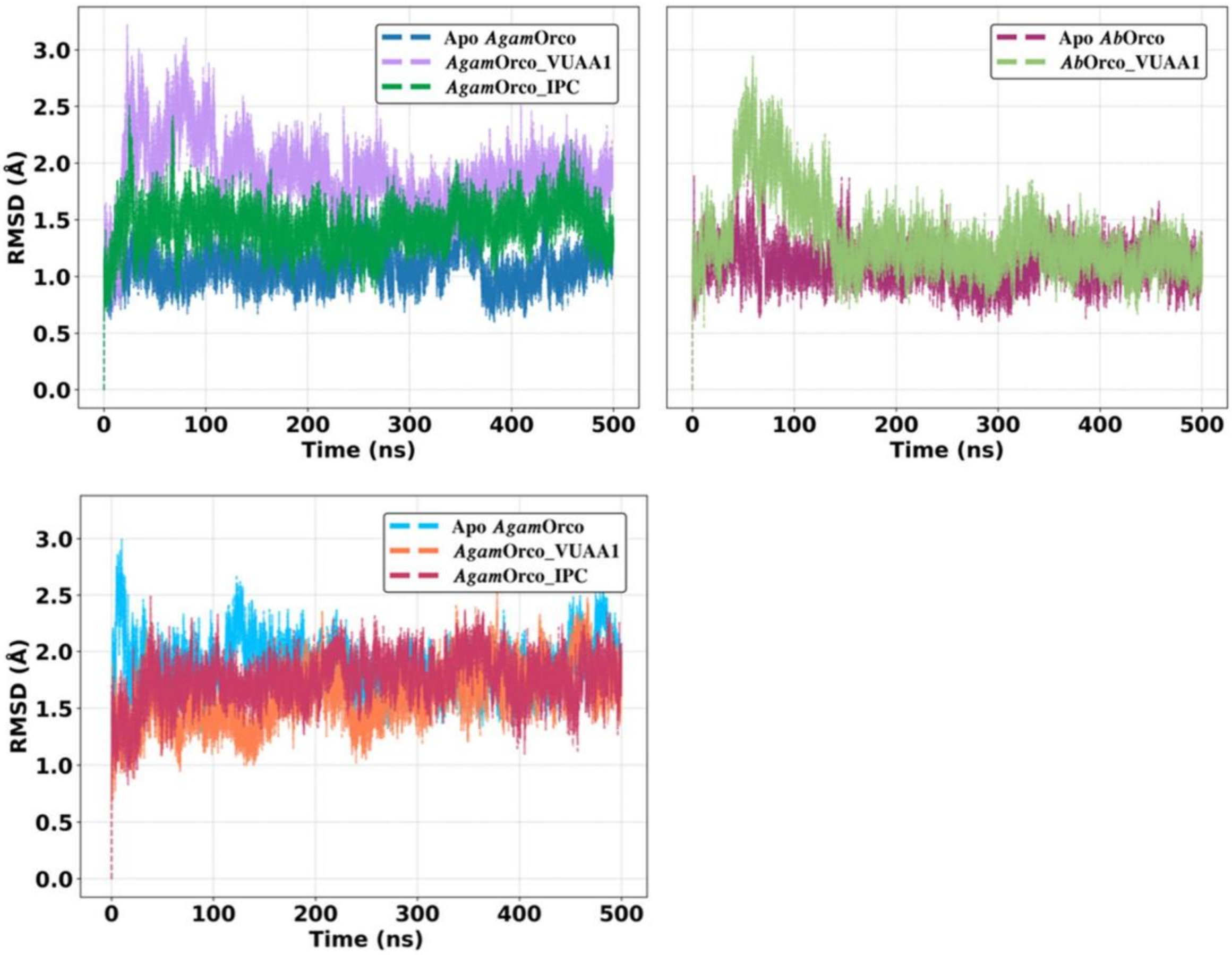

Figure S12. Pore RMSD profiles of the *Agam*Orco and *Ab*Orco homotetramer models over 500 ns of cMD simulations.

(a) TBM *Agam*Orco in the *apo* state and in complex with VUAA1 or IPC, (b) *Ab*Orco in the *apo* state and in complex with VUAA1, (c) AF3 *Agam*Orco in the *apo* state and in complex with VUAA1 or IPC.

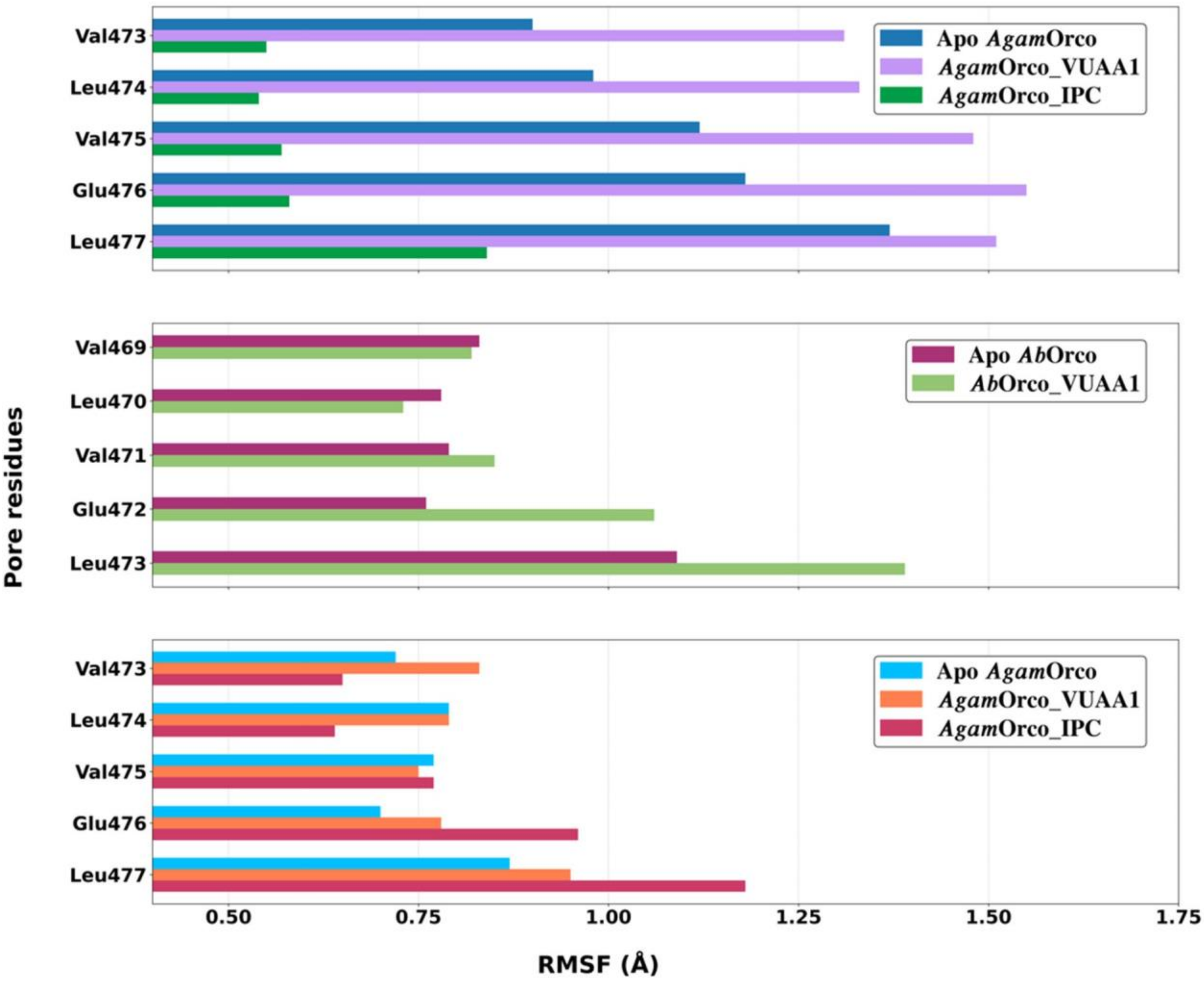

Figure S13. Pore RMSF profiles of the *Agam*Orco and *Ab*Orco homotetramer models over 500 ns of cMD simulations.

(a) TBM *Agam*Orco in the *apo* state and in complex with VUAA1 or IPC, (b) *Ab*Orco in the *apo* state and in complex with VUAA1, (c) AF3 *Agam*Orco in the *apo* state and in complex with VUAA1 or IPC.

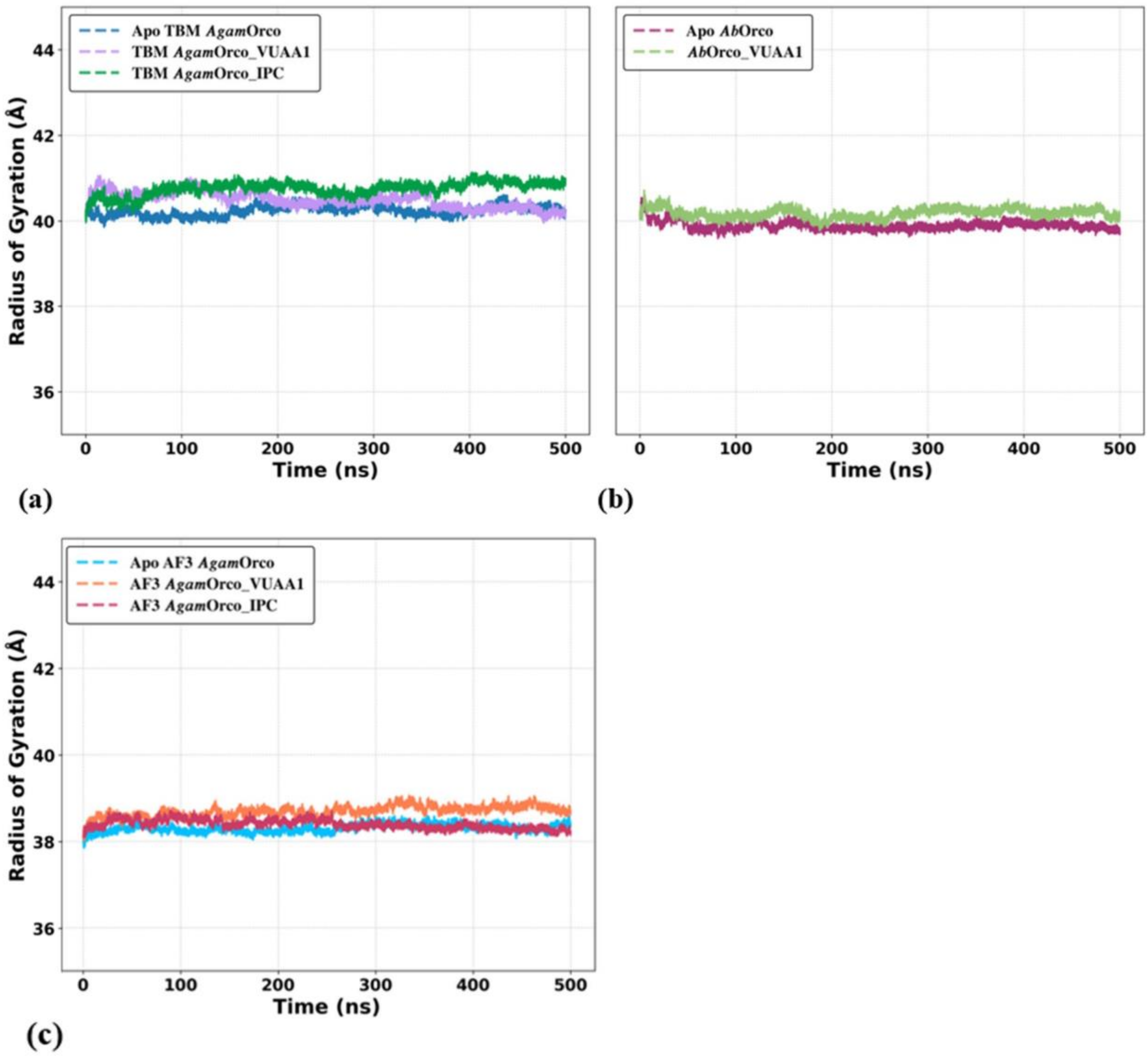

Figure S14. Radius of gyration profiles of the *Agam*Orco and *Ab*Orco homotetramer models over 500 ns of cMD simulations.

(a) TBM *Agam*Orco in the *apo* state and in complex with VUAA1 or IPC, (b) *Ab*Orco in the *apo* state and in complex with VUAA1, (c) AF3 *Agam*Orco in the *apo* state and in complex with VUAA1 or IPC.

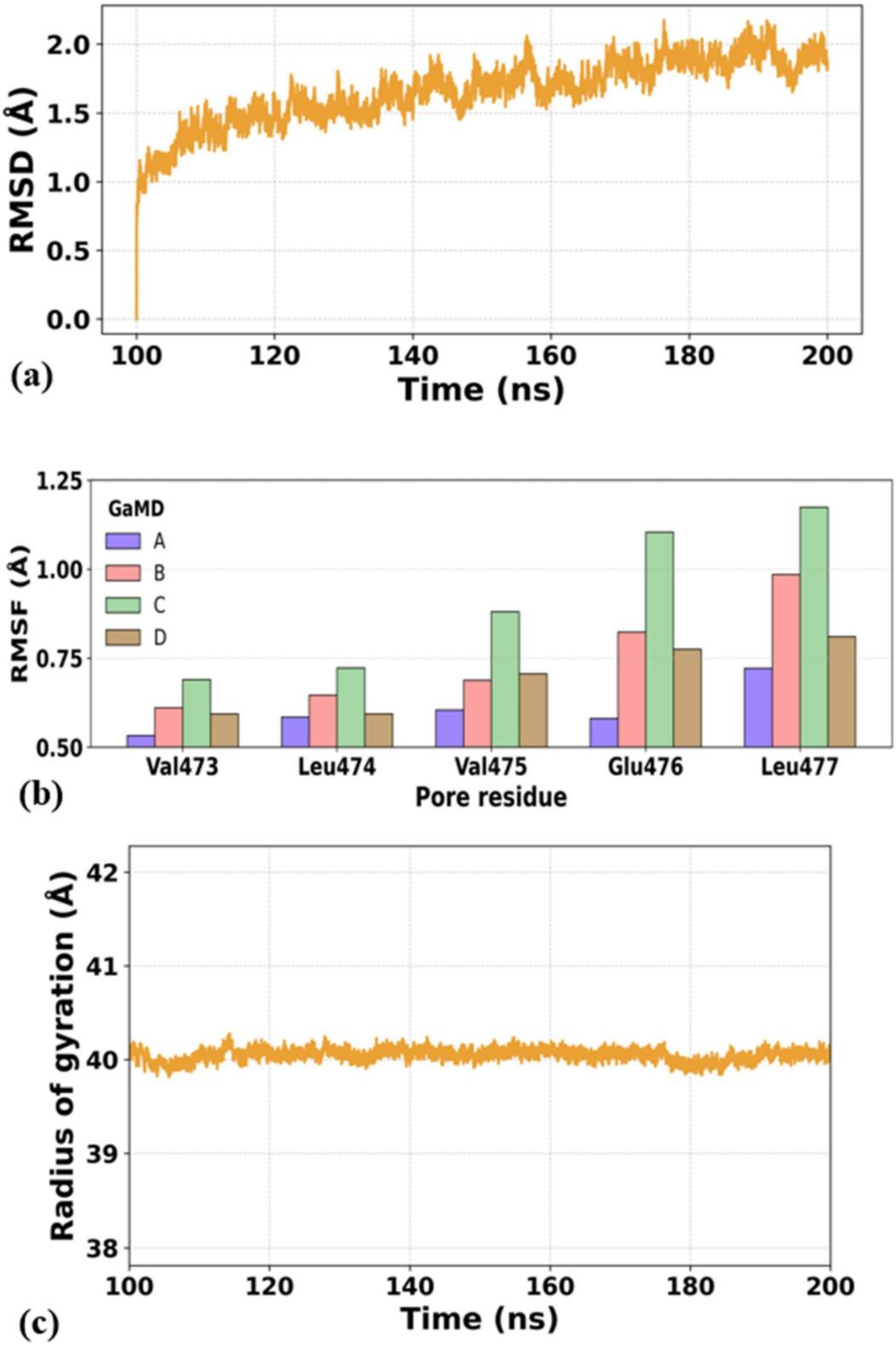

Figure S15. Analysis of *Agam*Orco complex with agonist VUAA1 in GaMD simulations.

(a) RMSD, (b) RMSF of the pore region in all 4 chains, and (c) Radius of Gyration.

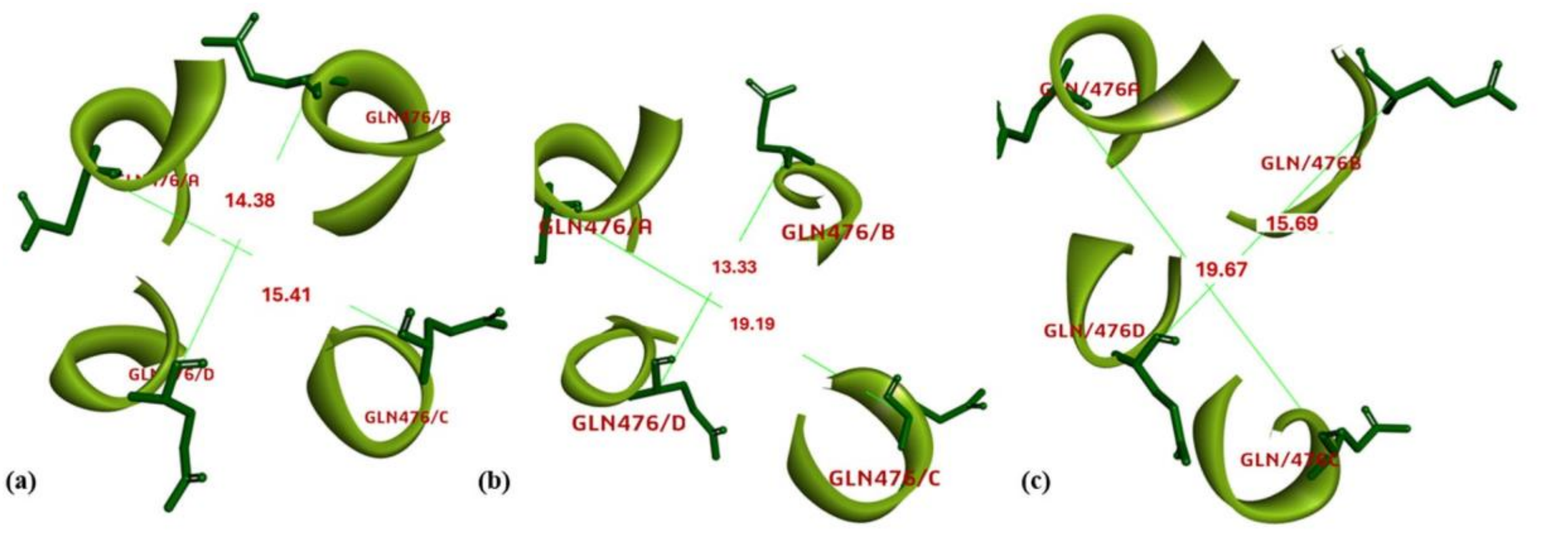

Figure S16. Inter-chain distance measurement of pore-gate residue Gln476 in *Agam*Orco homotetramer models post simulation.

Visualization of the inter-chain distances of the pore-gate residue Gln476 in *Agam*Orco homotetramer measured in (a) *Apo* *Agam*Orco homotetramer, (b) VUAA1 *Agam*Orco complex after 500 ns cMD simulation and (c) VUAA1 *Agam*Orco complex after GaMD simulation. The distances between chainpairs (AC and BD) are shown.

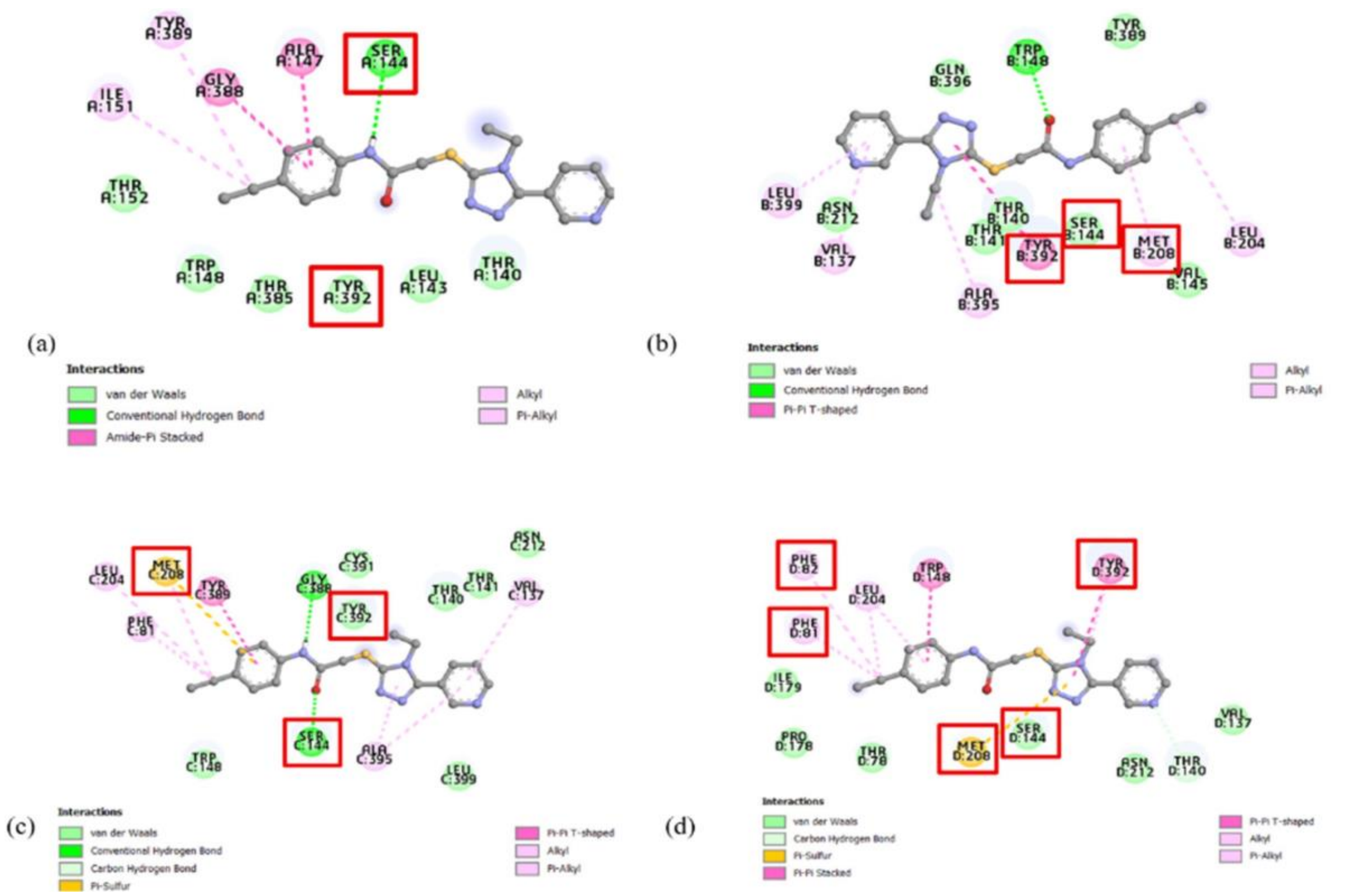

Figure S17. Interaction of VUAA1 in TBM *Agam*Orco model after 500 ns simulation.

Interaction of VUAA1 in TBM *Agam*Orco model in chains (a) A, (b) B, (c) C, and (d) D after 500 ns of simulation. The conserved binding pocket residues as reported [Pacalon *et al.* 2023] are boxed in red.

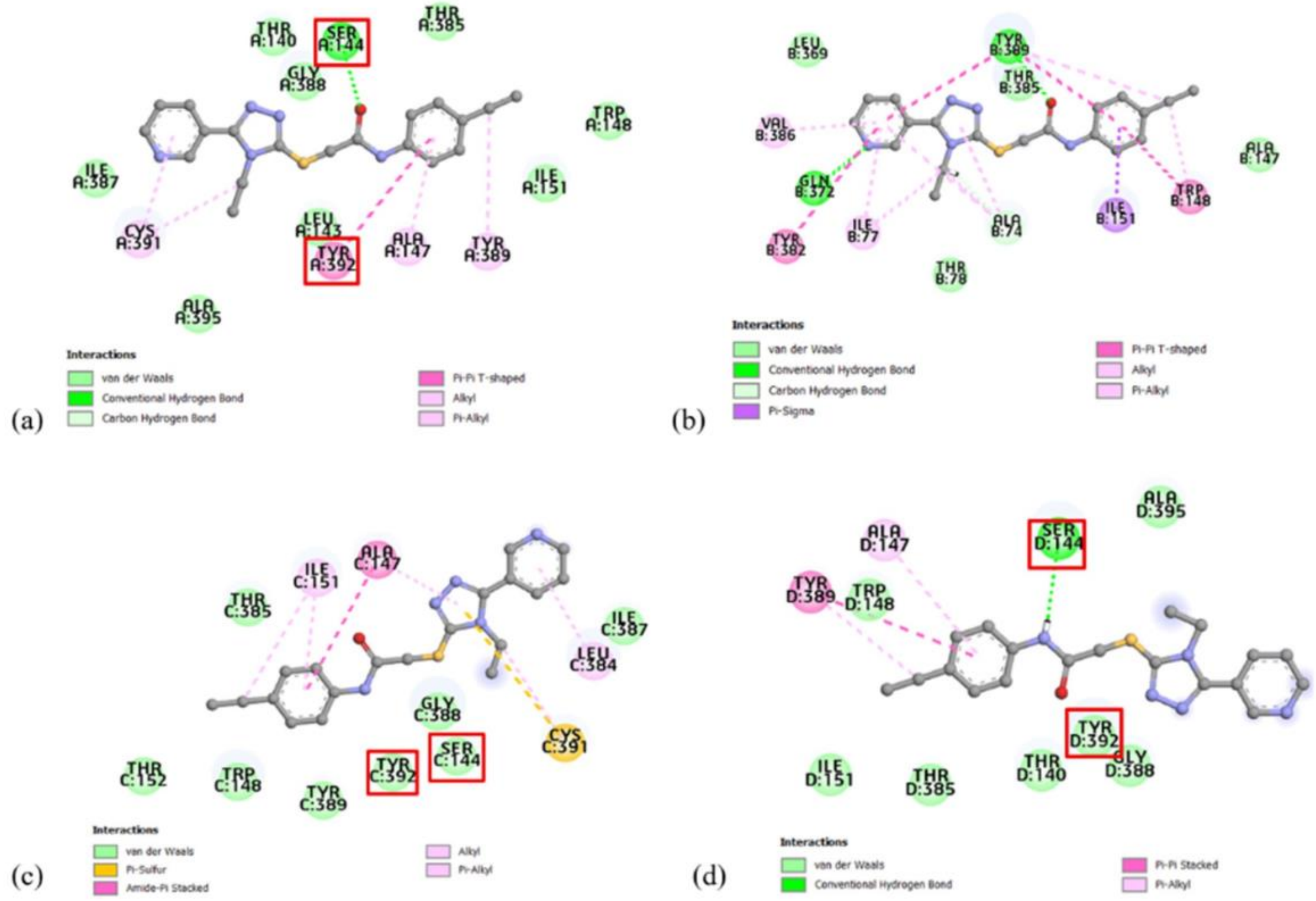

Figure S18. Interaction of VUAA1 in AF3 *Agam*Orco model after 500 ns simulation.

Interaction of VUAA1 in AF3 *Agam*Orco model in chains (a) A, (b) B, (c) C, and (d) D after 500 ns of simulation. The conserved binding pocket residues as reported [Pacalon *et al.* 2023] are boxed in red.

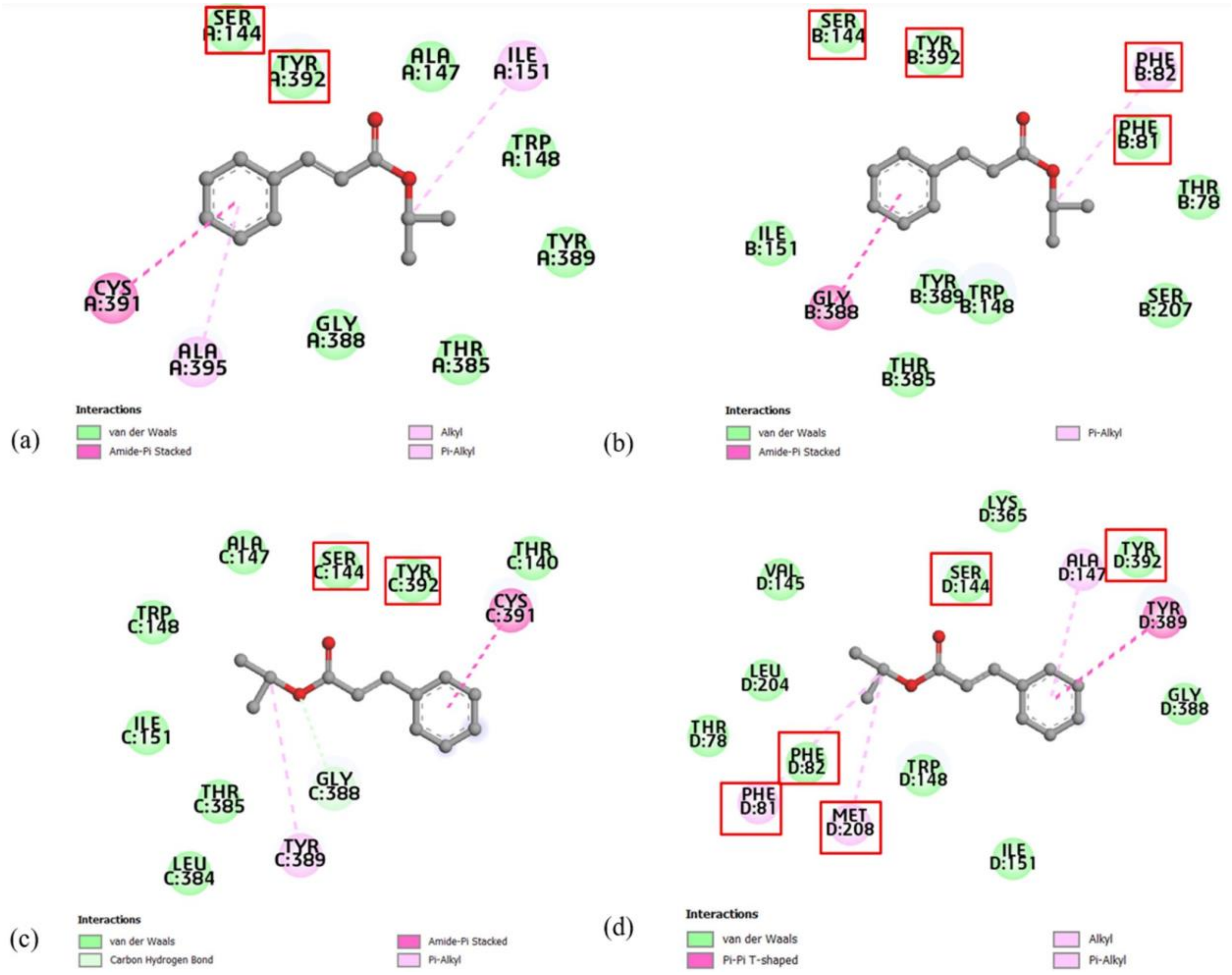

Figure S19. Interaction of IPC in TBM *Agam*Orco model after 500 ns simulation.

Interaction of IPC in TBM *Agam*Orco model in chains (a) A, (b) B, (c) C, and (d) D after 500 ns of simulation. The conserved binding pocket residues as reported [Pacalon *et al.* 2023] are boxed in red.

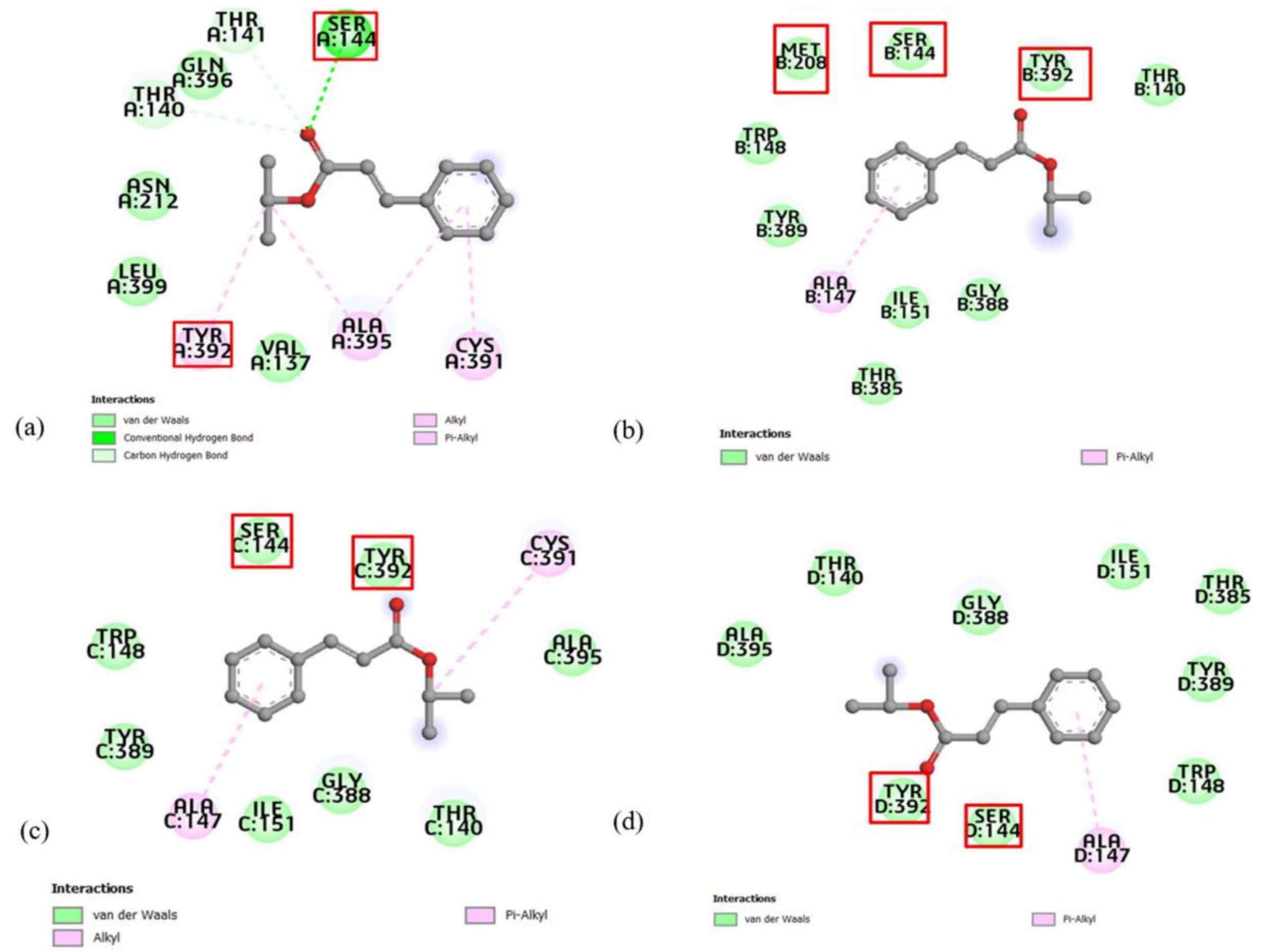

Figure S20. Interaction of IPC in AF3 *Agam*Orco model after 500 ns simulation.

Interaction of IPC in AF3 *Agam*Orco model in chains (a) A, (b) B, (c) C, and (d) D after 500 ns of simulation. The conserved binding pocket residues as reported [Pacalon *et al.* 2023] are boxed in red.

Table S4: Helical RMSD differences between the initial (1 ns) and final (500 ns) conformations of TBM *Agam*Orco complexes (mean values from three independent replicates).

Apo								
**Chain**	**Helix *S1***	**Helix *S2***	**Helix *S3***	**Helix *S4***	**Helix *S5***	**Helix *S6***	**Helix *S7a***	**Helix *S7b***
Chain A	0.61	1.19	1.23	0.90	0.87	0.86	0.39	0.60
Chain B	0.63	0.98	1.21	0.96	0.66	1.08	0.41	0.58
Chain C	0.58	1.08	1.45	0.92	0.96	1.12	0.44	0.58
Chain D	0.78	1.34	0.87	0.88	1.29	0.91	0.54	0.77
**VUAA1-bound**								
**Chain**	**Helix *S1***	**Helix *S2***	**Helix *S3***	**Helix *S4***	**Helix *S5***	**Helix *S6***	**Helix *S7a***	**Helix *S7b***
A	0.71	0.92	0.96	1.09	1.01	1.68	0.35	0.88
B	0.61	1.54	1.19	1.14	1.23	1.51	0.50	0.93
C	0.72	1.16	1.45	2.12	0.97	1.47	0.38	2.83
D	0.76	1.16	1.57	0.86	1.08	1.43	0.47	0.95
**IPC-bound**								
**Chain**	**Helix *S1***	**Helix *S2***	**Helix *S3***	**Helix *S4***	**Helix *S5***	**Helix *S6***	**Helix *S7a***	**Helix *S7b***
A	0.91	1.12	1.46	0.97	1.33	1.39	0.53	0.67
B	0.76	0.86	1.20	1.49	1.09	1.12	0.28	0.85
C	0.84	1.20	1.38	1.17	1.25	1.17	0.54	0.70
D	0.70	0.83	1.24	1.35	1.07	1.62	0.51	0.61

Table S5: Helix tilt and reorientation differences between the initial (1 ns) and final (500 ns) conformations of the *S7b* helices in TBM *Agam*Orco complexes (mean values from three replicates).

**Chain**	***Apo***		**VUAA1**		**IPC**	
	**Helix Tilt (°)**	**Helix reorientation(°)**	**Helix Tilt(°)**	**Helix reorientation(°)**	**Helix Tilt(°)**	**Helix reorientation(°)**
A	1.66	0.61	4.44	1.21	4.62	1.32
B	0.08	1.05	5.36	1.08	4.68	1.37
C	4.37	0.73	5.25	13.90	3.67	0.95
D	2.53	1.72	2.60	1.64	4.67	1.44

Table S6: Key changes in *S7b* hydrogen bond occupancy between the early (1-10 ns) and late (490-500 ns) phases of agonist-bound TBM *Agam*Orco complexes (mean values from three independent replicates).

Interaction type	Residues involved	Mean (early) (%)	Mean (late) (%)	Delta (%)	Lost/ formed
Inter-chain	LEU477(D)@O-GLN476(A)@NE2	24.1	13.4	-10.7	Reduced
Inter-chain	GLN476(A)@O-LYS478(D)@NZ	4.35	0	-4.35	Lost
Inter-chain	LEU477(A)@O-LYS478(B)@NZ	0	2.3	2.3	Formed
Intra-chain	LEU474(B)@O-LYS478(B)@N	18.3	0	-18.3	Lost
Intra-chain	VAL473(A)@O-GLN476(A)@N	19.9	0.8	-19.1	Reduced
Intra-chain	LEU474(A)@O-LEU477(A)@N	14.6	3.5	-11.1	Reduced
Intra-chain	VAL473(A)@O-LEU477(A)@N	10.5	27.9	17.4	Increased
Intra-chain	GLN476(C)@O-LYS478(C)@NZ	0.1	26	25.9	Formed

Table S7: Energy analysis after 500 ns in VUAA1 and IPC binding in the TBM and AF3 *Agam*Orco model and VUAA1 binding in *Ab*Orco model.

	**VUAA1**					
	**TBM *Agam*Orco**		***Ab*Orco**		**AF3 *Agam*Orco**	
**Chain**	**GB**	**PB**	**GB**	**PB**	**GB**	**PB**
A	-30.00	-6.50	-40.32	-11.73	-22.36	-4.53
B	-35.96	-4.87	-34.07	-8.85	-30.40	-1.55
C	-29.53	-4.72	-34.42	-7.27	-30.59	-3.41
D	-34.85	-4.95	-36.28	-5.40	-28.92	-3.19
	**IPC**					
	**TBM *Agam*Orco**		***Ab*Orco**		**AF3 *Agam*Orco**	
**Chain**	**GB**	**PB**	**GB**	**PB**	**GB**	**PB**
A	-21.76	-4.51	NA	NA	-17.70	-5.06
B	-22.99	-5.62	NA	NA	-17.93	-3.40
C	-21.75	-2.44	NA	NA	-19.20	-3.77
D	-21.74	-3.88	NA	NA	-18.59	-4.80

Note S4: The open pore closes following antagonist binding

To analyse the displacement of the antagonist IPC on the agonist VUAA1, two sets of simulations were conducted. In the first, the starting structure was the open pore conformation obtained from the cMD simulations with agonist binding. In the second, the starting structure was a specific open pore frame derived from the GaMD production simulation of the Orco homotetramer induced by agonist binding. In both cases, the initial open pore structure, after removal of the agonist VUAA1 in all chains, was used as a ligand-free open pore reference control, analogous to an *apo* state. IPC was then added to all four chains to assess how IPC binding influences pore closure and structural rearrangements in the agonist-bound open conformation. At the end of the cMD simulations, the antagonist rendered the starting open pore conformation closed (Figure S21).

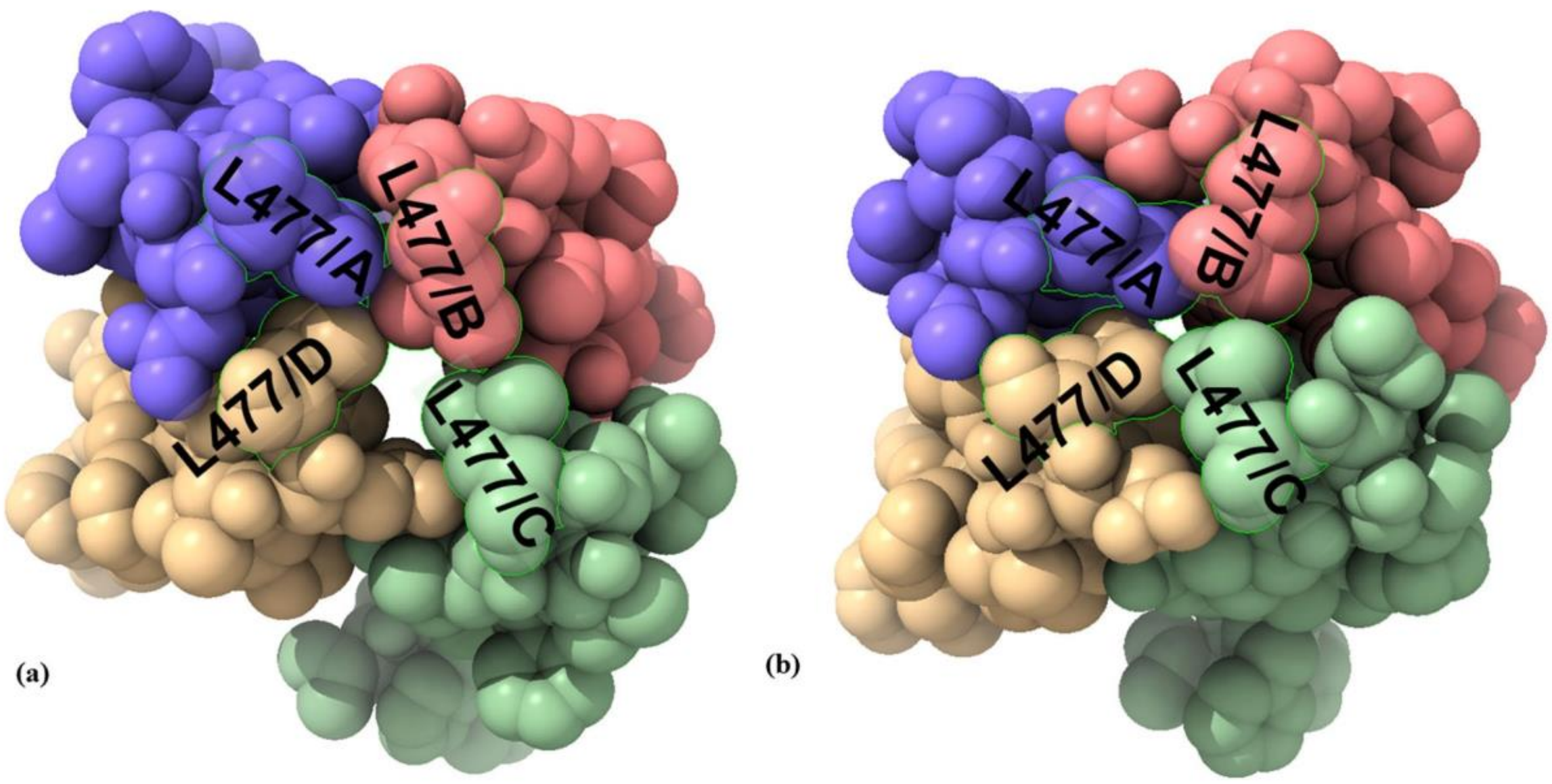

Figure S21. Open pore channel closes with antagonist binding in *Agam*Orco homotetramer.

(a) Open pore derived from cMD with agonist binding in *Agam*Orco homotetramer, (b) Pore closed post cMD when agonist is displaced by antagonist.

Table S8: Distance analysis (mean inter-chain distances in Å) after 200 ns and 500 ns of MD simulations of antagonist binding in the open pore conformations derived from cMD and GaMD.

**Open pore_cMD**					**Open pore_GaMD**				
Apo	**200 ns**		**500 ns**		***Apo***	**200 ns**		**500 ns**	
**Residue**	**A-C**	**B-D**	**A-C**	**B-D**	**Residue**	**A-C**	**B-D**	**A-C**	**B-D**
Val473	15.33	10.80	14.03	11.71	Val473	13.77	10.03	14.48	9.39
Leu474	17.05	15.19	16.39	15.41	Leu474	16.60	12.93	17.05	12.16
Val475	22.17	19.91	21.58	20.84	Val475	22.16	18.48	22.66	17.81
Gln476	18.87	15.63	17.77	18.02	Gln476	18.75	17.7	19.41	16.96
Leu477	13.17	11.19	13.12	14.77	Leu477	13.69	15.04	14.34	13.86
**With IPC**	**200 ns**		**500 ns**		**With IPC**	**200 ns**		**500 ns**	
**Residue**	**A-C**	**B-D**	**A-C**	**B-D**	**Residue**	A-C	B-D	A-C	B-D
Val473	12.31	11.37	12.06	11.19	Val473	15.45	8.36	14.84	8.92
Leu474	16.56	15.24	16.20	14.59	Leu474	17.64	11.48	16.19	12.26
Val475	20.68	20.29	20.27	19.62	Val475	23.16	17.23	21.86	17.55
Gln476	12.52	16.01	15.4	15.44	Gln476	19.59	16.08	19.40	13.61
Leu477	10.16	11.59	11.23	10.69	Leu477	13.83	13.89	13.63	13.60

Note S5: RMSD, RMSF and Rg analyses for agonist derived open pore conformation and antagonist-bound in open conformation in TBM *Agam*Orco HT models

Removing the agonist from the VUAA1 bound structure generated an *apo* structure in the open pore conformation. This model was subject to cMD. Independently, the antagonist IPC was docked to the open pore confirmation, followed by cMD simulations. In the cMD-derived system, RMSD fluctuations (Figure S22a) gradually increased, indicating that the open pore conformation is initially stable but underwent progressive structural relaxation. Antagonist binding appears to partially stabilise the pore compared to the *apo* state, though conformational changes still occur, consistent with the increased fluctuations observed in the ligand-free system. Similarly, the GaMD-derived system exhibited moderate RMSD fluctuations in the IPC-bound state, reflecting gradual pore narrowing, whereas the *apo* open pore system maintained lower RMSD values consistent with a stable open conformation (Figure S23a). Pore RMSD analysis in both systems indicated that the pore region remained dynamic throughout the simulation. In the cMD-derived system, IPC binding resulted in conformational changes consistent with narrowing, whereas the *apo* open pore maintained lower fluctuations (Figure S22b). In the GaMD-derived system, the IPC-bound pore underwent small dynamic changes but remained overall stable, while the *apo* open pore system displayed larger fluctuations, indicating higher structural variability (Figure S23b). In both systems, RMSF analysis showed higher fluctuations for the IPC-bound open pore compared to the *apo* open pore reference. In the cMD-derived system, higher RMSF values reflected increased local conformational changes during pore narrowing, rather than stabilising the pore (Figure S21c). The GaMD-derived system exhibited a similar trend, with the IPC-bound system showing higher fluctuation during gradual closure yet still maintaining a relatively stable overall structure than its *apo* (Figure S23c). Global compactness remained largely stable in both systems (Figure S22d, S23d). In the cMD-derived IPC-bound system, Rg was slightly higher but stable relative to the ligand-free open pore control, consistent with preserved overall structure during pore narrowing. Also, the post-MD binding energy analysis (Table S9) showed that IPC remained bound to the open pore *Agam*Orco conformation in both cMD- and GaMD-derived systems, which were weaker than those of the agonist-bound VUAA1 complex but comparable to the antagonist-bound IPC complex. In the cMD-derived system, ligands in chains A and B showed the strongest binding, with mean GB values of -25.71 and -25.37 kcal/mol, respectively, whereas the ligand in chain C showed the weakest binding at -14.19 kcal/mol, indicating less favourable stabilisation after agonist displacement. In the GaMD-derived system, IPC also showed moderate binding, suggesting that it occupies the orthosteric pocket and stabilises the receptor to some extent but does not maintain the active open pore conformation as effectively as the agonist, consistent with the observed shift of the pore toward a more constricted state after antagonist binding.

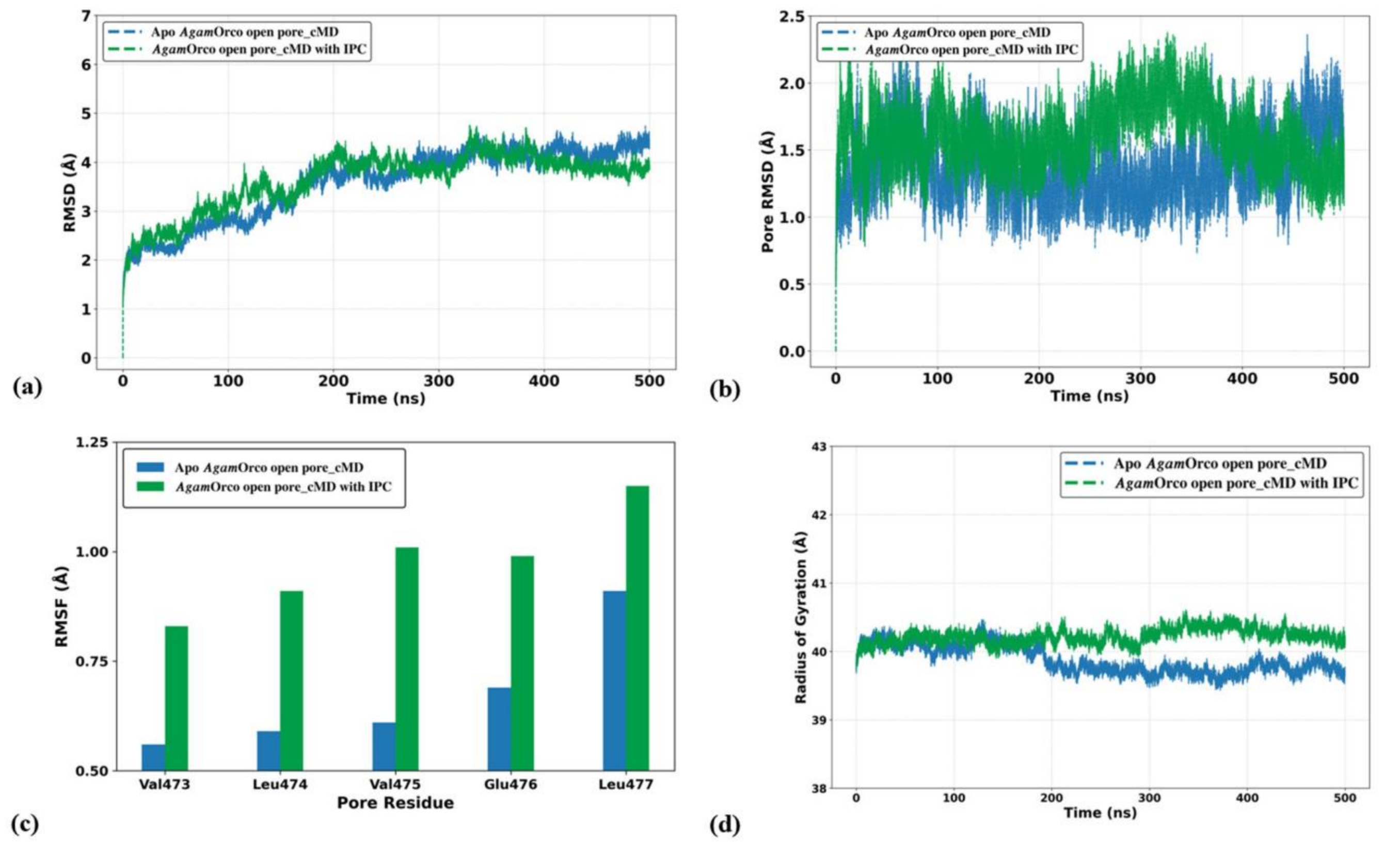

Figure S22. Post MD analysis of *Agam*Orco homotetramer simulations initiated from the cMD derived open pore conformation in *apo* and IPC-bound complex.

a. RMSD profiles, b. Pore-RMSD profiles, c. Pore-RMSF profiles, and d. Radius of gyration profiles.

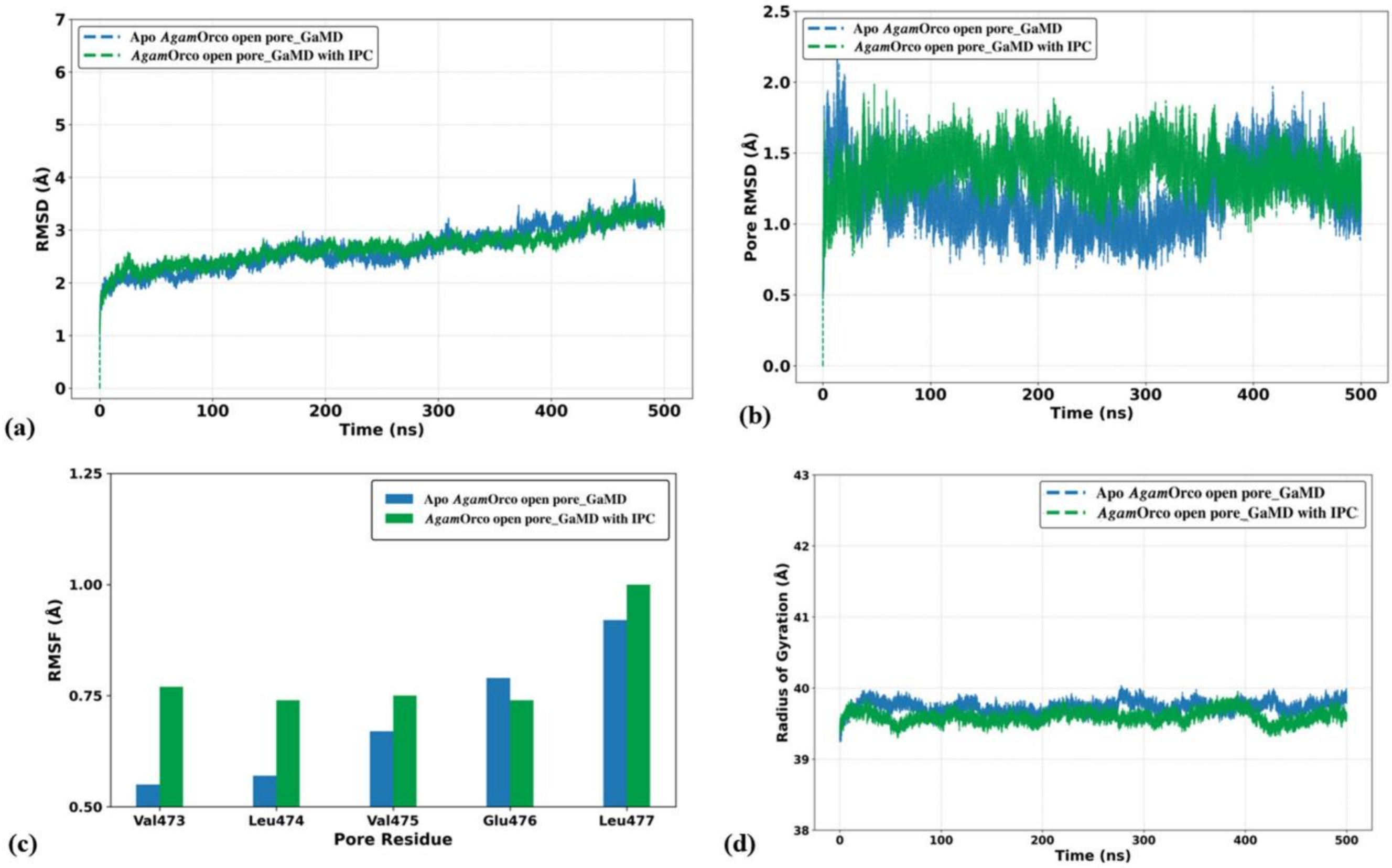

Figure S23. Post MD analysis of *Agam*Orco homotetramer simulations initiated from GaMD derived open pore conformation in *apo* and IPC-bound complex.

a. RMSD profiles, b. Pore-RMSD profiles, c. Pore-RMSF profiles, and d. Radius of gyration profiles.

Table S9. Energy analysis after 500 ns simulation in IPC binding in the cMD and GaMD derived open pore conformation in TBM *Agam*Orco model.

**TBM *Agam*Orco**	**Open pore from cMD with IPC**		**Open pore from GaMD with IPC**	
**Chain**	**GB**	**PB**	**GB**	**PB**
A	-25.39	-5.7	-25.4	-9.7
B	-25.37	-4.53	-23.46	-5.2
C	-14.86	-1.52	-20.94	-2.73
D	-21.4	-1.99	-26.63	-6.35

Note S6: The effect of agonist:antagonist concentration on pore dynamics in TBM *Agam*Orco model

The inter-chain distance of the pore-gate residues (Table S10) showed that the *Agam*Orco pore behaviour varied with ligand concentration, although none of these conditions resulted in pore opening.

RMSD profiles indicated rapid initial relaxation followed by stabilisation around 6-7 Å in all three ligand-ratio systems (Figure S24a). The antagonist-rich system exhibited larger fluctuations initially, before reaching equilibrium, whereas the agonist-rich system stabilised relatively earlier, suggesting that higher agonist concentration improved global stability. Pore RMSD analysis showed that the 1VUAA1:3IPC system had the largest pore-region fluctuations (Figure S24b). The 2VUAA1:2IPC system showed moderate fluctuations with gradual stabilisation, while the 3VUAA1:1IPC system exhibited fewer fluctuations, suggesting greater local pore stability under agonist-rich conditions. RMSF analysis showed higher pore-residue fluctuations in the antagonist-rich 1VUAA1:3IPC system, indicating reduced pore stability (Figure S24c). The balanced system showed moderate fluctuations, whereas the agonist-rich 3VUAA1:1IPC system displayed the lowest RMSF overall, indicating that agonists stabilise the pore conformation. Also, radius of gyration analysis indicated that global compactness was maintained across all systems (Figure S24d). The 3:1 agonist-rich system exhibited the strongest binding interactions (Table S11). Specifically, binding to chains A, B and C exhibited stronger binding energies (GB = -37.40, -27.91 and -33.64, respectively) compared to weaker binding observed in chain D (IPC) (GB = -21.24). In contrast, the 2:2 system showed moderate binding energies in chain A (VUAA1) exhibiting stronger binding energy (GB = -41.10) than in chain B (VUAA) (GB = -29.08), while Chain C (IPC) and Chain D (IPC) showed weaker binding energies of -21.30 and -21.88 kcal/mol, respectively. The 1:3 ratio (antagonist-rich) showed the weakest binding energy for chain A (IPC) (GB = -17.63), while chain B (IPC), chain C (IPC), and chain D (VUAA1) exhibited moderate binding energies of -25.62, -24.33, and -24.73 kcal/mol, respectively.

Table S10: Inter-chain pore-gate distances (mean distances in Å) at different ligand concentrations after 500 ns simulation in TBM *Agam*Orco model.

Residue	1 VUAA1: 3 IPC A-C	1 VUAA1: 3 IPC B-D	2 VUAA1: 2 IPC A-C	2 VUAA1: 2 IPC B-D	3 VUAA1: 1 IPC A-C	3 VUAA1: 1 IPC B-D
Val473	11.88	13.54	12.35	12.92	11.35	12.19
Leu474	13.97	15.95	14.01	15.45	15.47	15.68
Val475	19.16	20.89	19.4	19.77	20.09	19.91
Gln476	15.18	16.5	15.7	15.88	15.48	15.17
Leu477	9.88	11.86	10.58	10.16	10.79	9.8

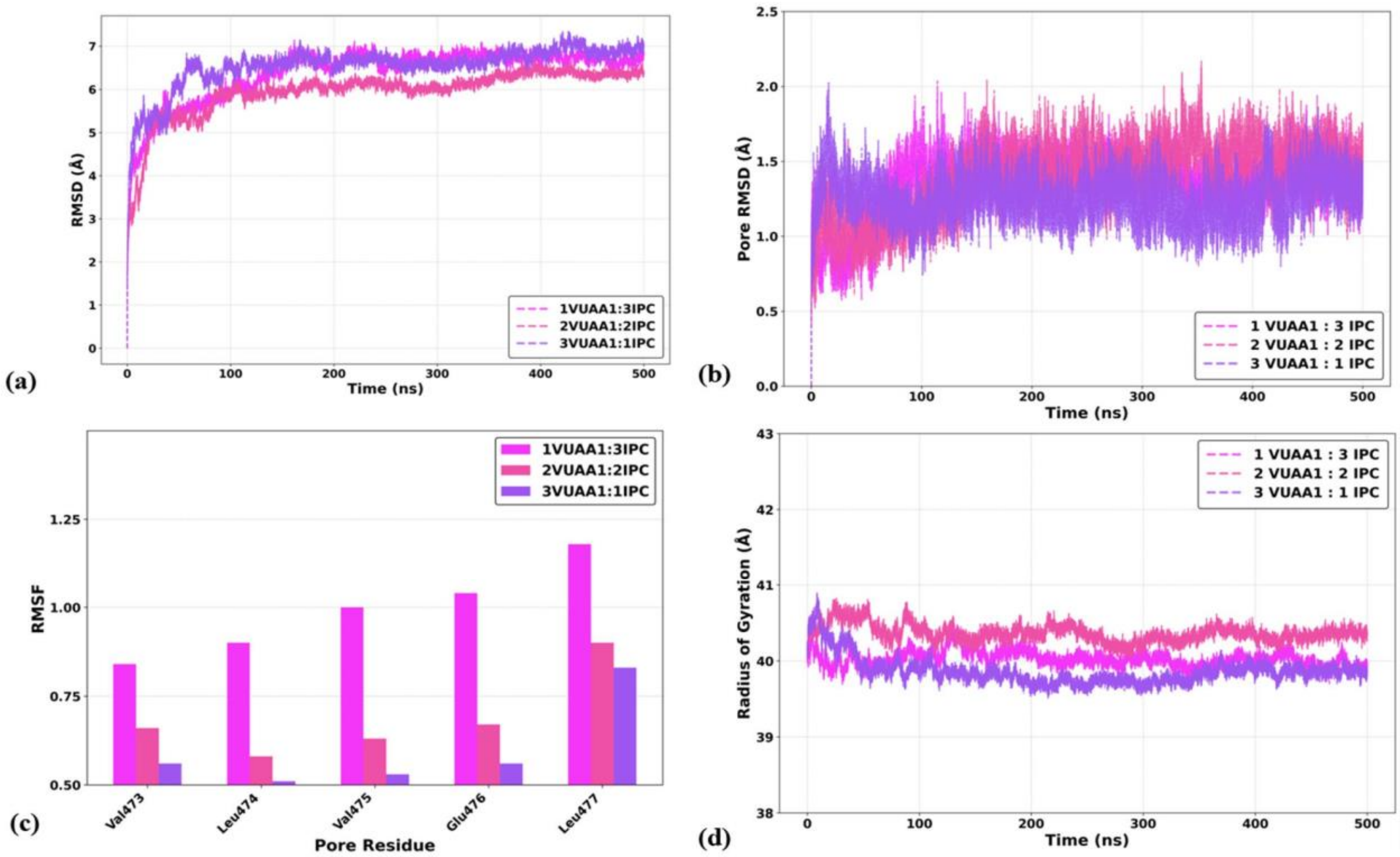

Figure S24. Post MD analysis of *Agam*Orco homotetramer simulations with different concentrations of VUAA1 and IPC.

a. RMSD profiles, b. Pore-RMSD profiles, c. Pore-RMSF profiles, and d. Radius of gyration profiles.

Table S11: Energy analysis after 500 ns in different ligand concentrations in TBM *Agam*Orco model.

	3VUAA1:1IPC			2VUAA1:2IPC			1VUAA1:3IPC	
**Chain/ Ligand**	**GB**	**PB**	**Chain/ Ligand**	**GB**	**PB**	**Chain/ Ligand**	**GB**	**PB**
A(VUAA1)	-37.4	-7.29	A(VUAA1)	-41.1	-9.79	A(IPC)	-17.63	-4.51
B(VUAA1)	-27.91	-3.36	B(VUAA1)	-29.08	-4.19	B(IPC)	-25.62	-3.21
C(VUAA1)	-33.64	-7.65	C(IPC)	-21.3	-6.09	C(IPC)	-24.33	-7.16
D(IPC)	-21.24	-2.77	D(IPC)	-21.88	0.92	D(VUAA1)	-24.73	-4.29

References

Angladon MA, Fosséprê M, Leherte L et al. Interaction of POPC, DPPC, and POPE with the μ opioid receptor: A coarse-grained molecular dynamics study. *PLoS One* 2019;14:e0213646. https://doi.org/10.1371/journal.pone.0213646

Butterwick JA, Del Mármol J, Kim KH et al. Cryo-EM structure of the insect olfactory receptor Orco. *Nature* 2018;560:447–52. https://doi.org/10.1038/s41586-018-0420-8

Case DA, Aktulga HM, Belfon K et al. *Amber*. University of California, San Francisco, 2022. https://ambermd.org/doc12/Amber22.pdf

He X, Man VH, Yang W et al. A fast and high-quality charge model for the next generation general AMBER force field. *J Chem Phys* 2020;153:114502. https://doi.org/10.1063/5.0019056

Jo S, Kim T, Iyer VG et al. CHARMM-GUI: a web-based graphical user interface for CHARMM. *J Comput Chem* 2008;29:1859–65. https://doi.org/10.1002/jcc.20945

Maier JA, Martinez C, Kasavajhala K et al. ff14SB: Improving the Accuracy of Protein Side Chain and Backbone Parameters from ff99SB. *J Chem Theory Comput* 2015;11:3696–713. https://doi.org/10.1021/acs.jctc.5b00255

Moreno MJ, Teles Martins PA, Bernardino EF et al. Characterization of the Lipidome and Biophysical Properties of Membranes from High Five Insect Cells Expressing Mouse P-Glycoprotein. *Biomolecules* 2021;11:426. https://doi.org/10.3390/biom11030426

Pacalon J, Audic G, Magnat J et al. Elucidation of the structural basis for ligand binding and translocation in conserved insect odorant receptor co-receptors. *Nat Commun* 2023;14:8182. https://doi.org/10.1038/s41467-023-44058-5

Wang J, Arantes PR, Bhattarai A et al. Gaussian accelerated molecular dynamics (GaMD): principles and applications. *Wiley Interdiscip Rev Comput Mol Sci* 2021;11:e1521. https://doi.org/10.1002/wcms.1521

Waterhouse A, Bertoni M, Bienert S et al. SWISS-MODEL: homology modelling of protein structures and complexes. *Nucleic Acids Res* 2018;46:W296–303. https://doi.org/10.1093/nar/gky427

Wu EL, Cheng X, Jo S et al. CHARMM-GUI Membrane Builder toward realistic biological membrane simulations. *J Comput Chem* 2014;35:1997–2004. https://doi.org/10.1002/jcc.23702

Yusuf M, Destiarani W, Firdaus ARR et al. Residual Interactions of LL-37 with POPC and POPE:POPG Bilayer Model Studied by All-Atom Molecular Dynamics Simulation. *Int J Mol Sci* 2022;23:13413. https://doi.org/10.3390/ijms232113413
